# Supplementary material for: TGF-β1-based restoration of sodium iodide symporter expression in radioiodine-refractory differentiated thyroid cancer via engineered MSCs
Source: Mol Ther. 2025 Oct 17;33(12):6130–45. doi: 10.1016/j.ymthe.2025.10.033 (PMC12703174; doi:10.1016/j.ymthe.2025.10.033)
Supplement: Document S1. Figures S1 and S2 [file mmc1.pdf]

YMTHE, Volume *33*

## Supplemental Information

# TGF-β1-based restoration of sodium iodide symporter expression in radioiodine-refractory differentiated thyroid cancer via engineered MSCs

**Yang Han, Viktoria F. Koehler, James Nagarajah, Kathrin A. Schmohl, Christina Stauss, Nathalie Schwenk, Rebekka Spellerberg, Carolin Kitzberger, Joerg Kumbrink, Christian Zach, Katja Steiger, John C. Morris, Wolfgang A. Weber, Peter Bartenstein, Sibylle I. Ziegler, Peter J. Nelson, and Christine Spitzweg**

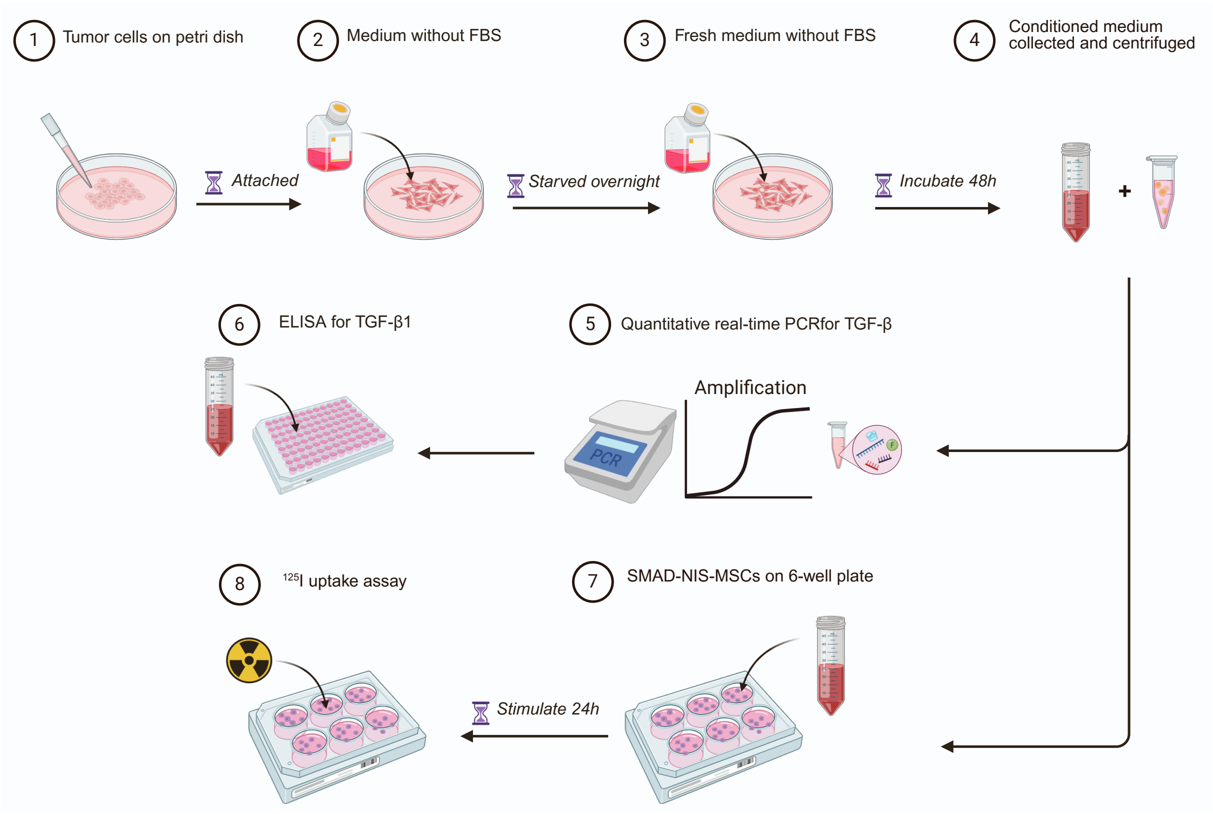


***Figure S1. TGF-β expression in thyroid cancer cell lines and stimulation of RAI uptake in SMAD-NIS-MSCs by tumor CM.*** *Illustration of the experimental design.*

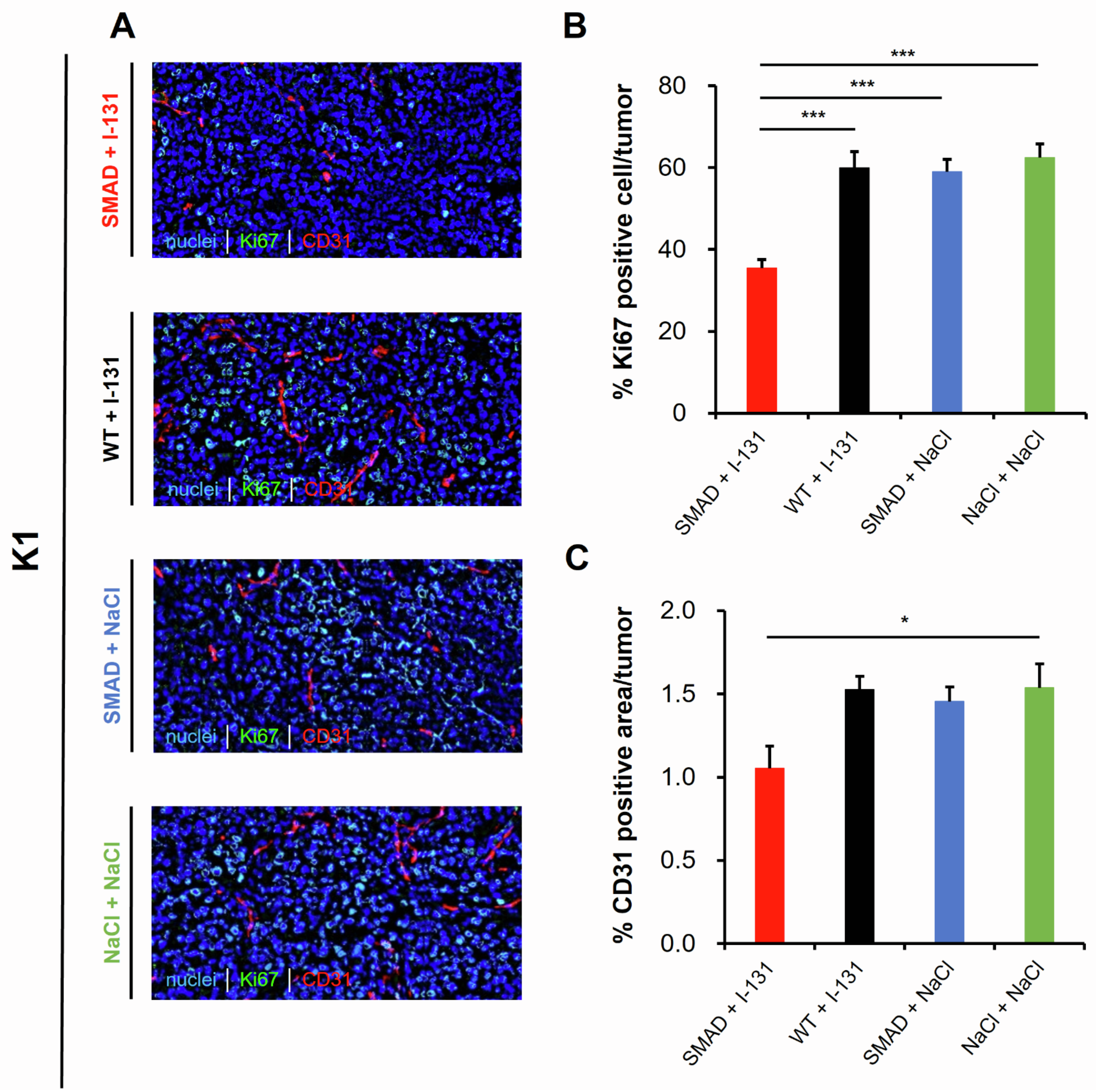
A
B
C
K1
SMAD + I-131
WT + I-131
SMAD + NaCl
NaCl + NaCl
nuclei | Ki67 | CD31
% Ki67 positive cell/tumor
% CD31 positive area/tumor
***
*
SMAD + I-131
WT + I-131
SMAD + NaCl
NaCl + NaCl

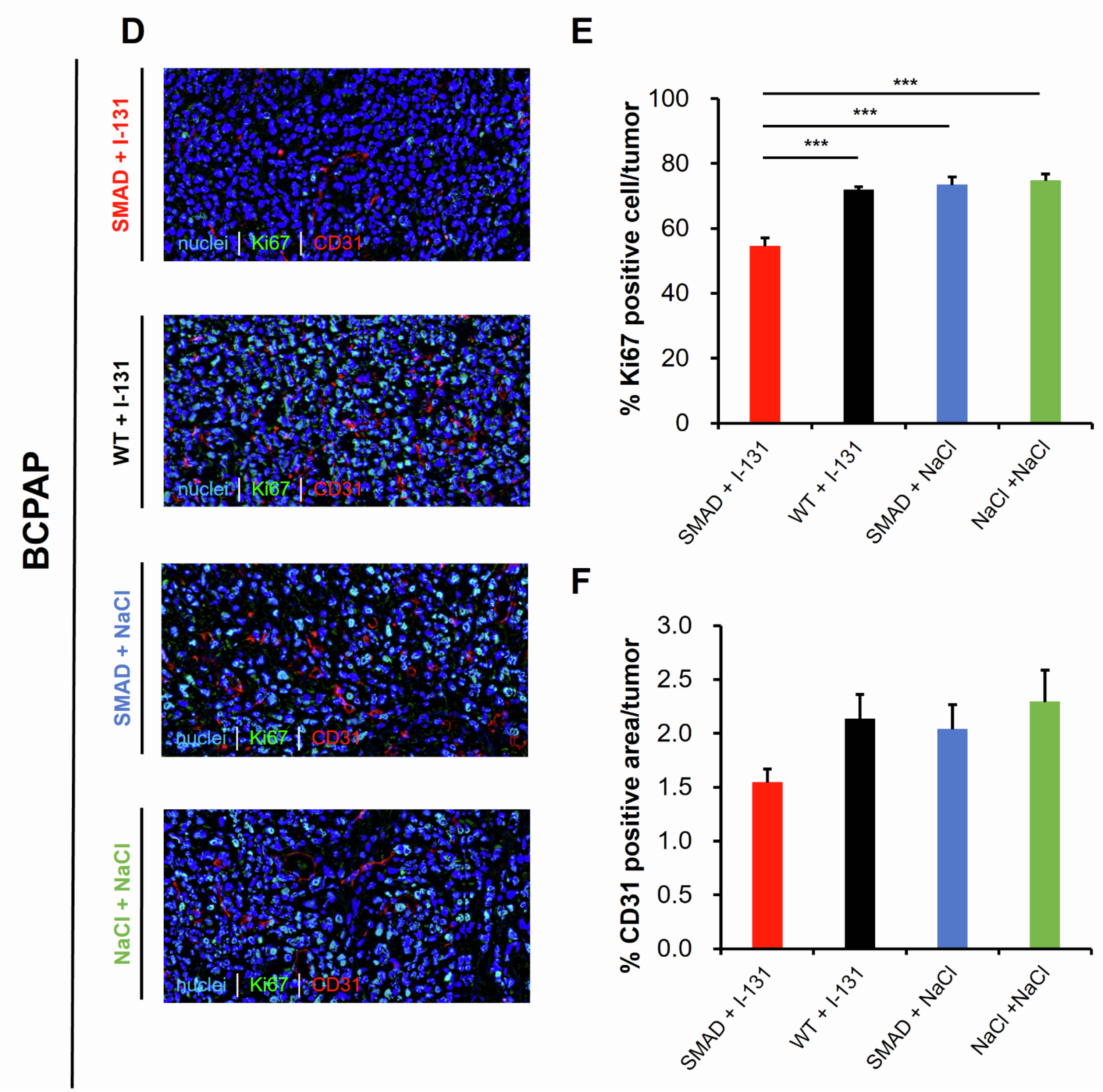


***Figure S2. Ex vivo analysis of tumor cell proliferation and vascularization.*** *Tumor cell proliferation was assessed by Ki67 immunofluorescence staining (green) and blood vessel density by CD31 staining (red) on frozen tumor sections **(A, D)**. The staining revealed significantly decreased proliferation as well as reduced blood vessel density in K1 tumors **(B, C)** and BCPAP tumors **(E, F)** treated with SMAD-NIS-MSCs*

*followed by $^{131}$I treatment as compared to the control tumors (mean ± SEM; *p<0.05, ***p<0.001). One representative image is shown each (20× magnification).*
