# Supplementary material for: TGF-β1-based restoration of sodium iodide symporter expression in radioiodine-refractory differentiated thyroid cancer via engineered MSCs
Source: Mol Ther. 2025 Oct 17;33(12):6130–45. doi: 10.1016/j.ymthe.2025.10.033 (PMC12703174; doi:10.1016/j.ymthe.2025.10.033)
Supplement: Document S2. Article plus supplemental information [file mmc2.pdf]

Molecular Therapy
Original Article

TGF-β1-based restoration of sodium iodide symporter expression in radioiodine-refractory differentiated thyroid cancer via engineered MSCs

Yang Han,[1,12] Viktoria F. Koehler,[1,12] James Nagarajah,[2,3] Kathrin A. Schmohl,[1] Christina Stauss,[1] Nathalie Schwenk,[1] Rebekka Spellerberg,[1] Carolin Kitzberger,[4] Joerg Kumbrink,[5] Christian Zach,[6] Katja Steiger,[7,8,9] John C. Morris,[10] Wolfgang A. Weber,[4,8,9] Peter Bartenstein,[6] Sibylle I. Ziegler,[6] Peter J. Nelson,[1] and Christine Spitzweg[1,8,9,11]

[1]Department of Internal Medicine IV and Comprehensive Cancer Center, LMU University Hospital of Munich, LMU Munich, Munich, Germany; [2]Department of Radiology and Nuclear Medicine, Radboud University Medical Center, Nijmegen, the Netherlands; [3]Röntgeninstitut, Düsseldorf, Germany; [4]Department of Nuclear Medicine, School of Medicine, TUM University Hospital, Klinikum Rechts der Isar, Technical University of Munich, Munich, Germany; [5]Institute of Pathology, Faculty of Medicine, LMU Munich, Munich, Germany; [6]Department of Nuclear Medicine, University Hospital of Munich, LMU Munich, Munich, Germany; [7]Comparative Experimental Pathology, Institute of Pathology, School of Medicine, TUM University Hospital, Klinikum Rechts der Isar, Technical University of Munich, Munich, Germany; [8]Bavarian Cancer Research Center, Munich, Germany; [9]German Cancer Consortium (DKTK), Partner Site Munich, a Partnership between DKFZ and LMU and TUM Klinikum, Munich, Germany; [10]Divisions of Endocrinology, Diabetes, Metabolism, and Nutrition and Medical Oncology, Mayo Clinic, Rochester, MN, USA; [11]Division of Endocrinology, Diabetes, Metabolism, and Nutrition, Mayo Clinic, Rochester, MN, USA

The sodium iodide symporter (NIS) mediates iodide uptake into thyroid follicular cells, providing the basis for radioiodine (RAI) imaging and therapy of differentiated thyroid cancer (DTC). Loss of functional *NIS* expression leads to RAI-refractory disease. Restoration of RAI uptake via *NIS* delivery into tumors using mesenchymal stem cells (MSCs) represents a therapeutic strategy for RAI-refractory DTC. This study exploits transforming growth factor β (TGF-β) biology in RAI-refractory BRAF^{V600E}-mutant papillary thyroid cancer (PTC) to selectively drive *NIS* transgene expression in engineered MSCs using a synthetic TGF-β1-inducible SMAD-responsive promoter (SMAD-NIS-MSCs) to reestablish RAI accumulation. The ^{125}I uptake assay confirmed stimulation of functional NIS expression in SMAD-NIS-MSCs through TGF-β1 released from BRAF^{V600E}-mutant PTC cell lines (BCPAP, K1) from co-culture or by incubation with tumor-conditioned medium. Chemotaxis assays showed directed chemotaxis of MSCs toward BCPAP- and K1-tumor-conditioned medium. SMAD-NIS-MSCs applied intravenously to mice harboring BCPAP or K1 xenografts followed by ^{123}I scintigraphy demonstrated tumor-specific MSC recruitment and RAI accumulation. Application of SMAD-NIS-MSCs followed by ^{131}I demonstrated a significant delay in tumor growth with prolonged survival. We demonstrate re-establishment of NIS-mediated RAI therapy in RAI-refractory BRAF^{V600E}-mutant PTC tumors using MSC-mediated *NIS* gene delivery driven by their TGF-β1 biology.

INTRODUCTION

One of the hallmarks of advanced differentiated thyroid cancer (DTC) is reduced or diminished functional expression of the sodium iodide symporter (NIS) limiting the efficacy of radioiodine (RAI) therapy, the first choice systemic therapy for metastatic DTC.[1,2] NIS as an intrinsic transmembrane glycoprotein is expressed at the basolateral membrane of thyroid follicular cells mediating transport of iodide into the thyroid gland and other NIS-expressing organs. This physiologic function of NIS provides the basis for diagnostic imaging and treatment of DTC and its metastases by application of RAI.[3,4] RAI-refractory disease in DTC develops when functional NIS expression is decreased and/or when its membrane targeting is diminished.[2]

Important molecular mechanisms underlying the loss of functional *NIS* expression in RAI-refractory DTC have been elucidated.[2] Aberrant activation of the mitogen-activated protein kinase (MAPK) pathway driven mainly by the oncogene *BRAF*^{V600E}, receptor tyrosine kinase fusions (e.g., *RET* or *NTRK* fusions), and *RAS* mutations play a central role in the loss of expression of iodide-metabolizing genes such as *NIS* or the impairment of its membrane targeting, a prerequisite for functional NIS activity.[5,6]

The *BRAF*^{V600E} mutation is the most frequent genetic event seen in recurrent RAI-refractory PTC, in a subset of poorly differentiated TCs (PDTCs) as well as anaplastic TC (ATC) that derives from

Received 21 January 2025; accepted 14 October 2025;
https://doi.org/10.1016/j.ymthe.2025.10.033.

[12]These authors contributed equally

Correspondence: Christine Spitzweg, LMU University Hospital of Munich, LMU Munich Department of Internal Medicine IV, Marchioninistrasse 15, 81377 Munich, Germany.
E-mail: christine.spitzweg@med.uni-muenchen.de

 © 2025 The Authors.
Published by Elsevier Inc. on behalf of The American Society of Gene and Cell Therapy.
This is an open access article under the CC BY license (http://creativecommons.org/licenses/by/4.0/).

preexisting PTCs.[7–9] An autocrine loop involving transforming growth factor β (TGF-β) secretion induced by $BRAF^{V600E}$ has been shown to repress membrane-associated NIS expression through TGF-β/SMAD signaling.[10,11] TC, including PTC, is associated with high levels of TGF-β[12–17] that can in turn inhibit iodide uptake and NIS expression in thyroid follicular cells.[18,19]

Pilar Santisteban's group have demonstrated that the activating *$BRAF^{V600E}$* mutation drives enhanced secretion of TGF-β.[11] In addition, $BRAF^{V600E}$-induced TGF-β secretion was shown to reduce NIS-mediated iodide accumulation in thyroid cells that could be rescued upon SMAD7 inhibition.[10] Although this mechanism was shown to be mitogen-activated protein kinase kinase/extracellular signal-regulated kinase (MEK/ERK) independent, secreted TGF-β was described to cooperate with MEK/ERK signaling in $BRAF^{V600E}$-induced cell migration and invasion and epithelial-mesenchymal transition (EMT).[10] Consistent with these findings, TGF-β, TβRII, and pSMAD2 were demonstrated to be overexpressed in human PTC samples where high TGF-β/SMAD activity was shown to be associated with PTC invasion, nodal metastases, and BRAF status.[10] Of note, TGF-β expression was found to be stronger in the invasive regions of PTC versus the central regions.[10] Using a $BRAF^{V600E}$-induced PTC mouse model (*Tg-Braf*), Knauf et al. have shown that TGF-β plays an important role in PTC-PDTC progression.[20] Oncogenic BRAF-induced tumor initiation was found to render thyroid cells susceptible to TGF-β-driven EMT, a process shown to be MAPK dependent.[20] In a more recent study, the same group confirmed that $BRAF^{V600E}$-driven PTCs in mice (TPO-Cre/LSL-$Braf^{V600E}$) and humans show a marked increase in SMAD transcriptional output that could be reduced by treatment with MEK or RAF inhibitors.[21] Thus, activation of the MAPK pathway by $BRAF^{V600E}$ can promote SMAD activation through TGF-β signaling.[21]

These data demonstrate that an autocrine TGF-β loop is a key mechanism through which $BRAF^{V600E}$ induces NIS repression and promotes EMT and invasion in DTC, with increasing evidence suggesting that the activation of SMAD signaling induced through TGF-β or other TGF-β family ligands plays a critical role in RAI resistance and tumor invasion during the progression of $BRAF^{V600E}$-driven DTC. These findings point to a potential role for TGF-β as a therapeutic target for restoring NIS expression in RAI-refractory DTC and dedifferentiated TC.

In the present study, we aimed to re-establish responsiveness to RAI therapy in RAI-refractory DTC by using the high levels of TGF-β present in these tumors to re-induce NIS-mediated RAI uptake via tumor stroma-selective *NIS* gene delivery using a synthetic SMAD-responsive promoter to drive *NIS* expression in engineered mesenchymal stem cells (SMAD-NIS-MSCs) as delivery vehicles to the tumor microenvironment. Tumors such as DTC are thought to be seen by the body as chronic wounds that drive constant tissue remodeling with associated robust recruitment of MSCs.[22,23] The general tumor tropism of MSCs has been adapted for therapeutic approaches where adoptively applied genetically engineered MSCs have been used to deliver therapeutic genes deep into critical tumor environments, with very promising results in preclinical and clinical studies.[23–33]

The use of *NIS* as a potent and well-characterized theranostic gene in this context allows detailed non-invasive *in vivo* tracking of MSC homing and engraftment at the tumor site as well as the effective application of radionuclides, such as ^{131}I or ^{188}Re,[24,33–44] eliciting a therapeutic response in tumor stromal cells and adjacent tumor cells through their crossfire effect.[45]

The homing of adoptively applied MSCs to normal tissue as part of normal homeostasis represents a source of potential negative side effects of MSC-driven tumor therapy. This can be addressed through the use of gene promoters that are selectively induced within the engineered MSCs in response to exogenous signals found within tumor microenvironments to drive a more tumor-selective transgene expression,[34,36–38,40,42,45] thereby offering the possibility of tailoring NIS therapy approaches to the micromilieu of any individual tumor.

Based on the essential role of TGF-β in tumor biology, we previously established a synthetic TGF-β1/SMAD-responsive promoter to control and focus *NIS* transgene expression within tumors using engineered MSCs (SMAD-NIS-MSCs). We have shown that these cells can be actively recruited to growing tumors and that they induce functional NIS expression in response to the TGF-β present in the tumor milieu.[40,42]

As emerging evidence suggests that stimulation of SMAD signaling induced by TGF-β or other TGF-β family ligands plays a central role in the downregulation of *NIS* expression during progression of $BRAF^{V600E}$-mutant DTC, we proposed the restoration of responsiveness to RAI by co-opting the high levels of TGF-β family ligands in these tumors to re-induce NIS-mediated RAI uptake using SMAD-NIS-MSCs.

In the present study, we show that after adoptively applying SMAD-NIS-MSCs in $BRAF^{V600E}$-mutant PTC cell xenografts, MSC biodistribution and biological targeting of *NIS* expression to the tumor stroma can be evaluated by ^{123}I scintigraphy when using *NIS* as a reporter gene. Using *NIS* in its function as a therapy gene, therapeutic efficacy was demonstrated after ^{131}I application and systemic SMAD-NIS-MSC-mediated *NIS* transgene delivery.

RESULTS

Measurement of TGF-β mRNA and protein levels in different $BRAF^{V600E}$-mutant TC cell lines

The experimental design is illustrated in Figure S1. To characterize TGF-β expression in $BRAF^{V600E}$-mutant TC cell lines, the three TC cell lines BCPAP, K1, and BHT101 were evaluated for mRNA expression of TGF-β1, TGF-β2, and TGF-β3 (Figure 1A). All cell lines showed TGF-β1 expression, with the highest levels seen in BCPAP cells (Figure 1A). For TGF-β2, mRNA expression was detected in all three cell lines (Figure 1A). TGF-β3 was largely absent in all three TC cell lines tested (Figure 1A). To verify TGF-β1

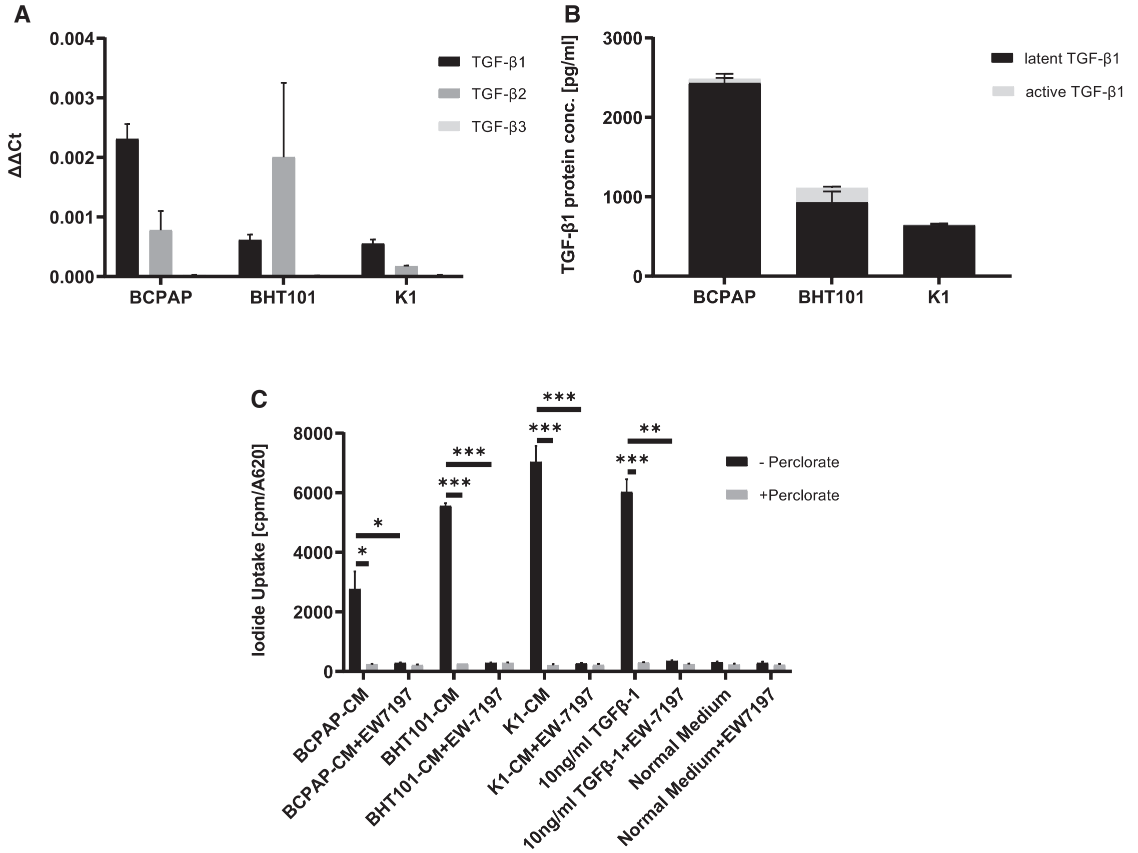

Figure 1. TGF-β expression in TC cell lines and stimulation of RAI uptake in SMAD-NIS-MSCs by tumor CM

(A) Quantitative real-time PCR analysis of TGF-β1, -β2, and -β3 mRNA extracted from BCPAP, BHT101, and K1. (B) TGF-β1 protein levels in CM derived from TC cell lines were assessed by ELISA. (C) ^{125}I uptake studies demonstrated significant stimulation of NIS-specific, perchlorate-sensitive iodide uptake in SMAD-NIS-MSCs stimulated with BCPAP-CM, BHT101-CM, and K1-CM as compared with SMAD-NIS-MSCs incubated with normal medium. Treatment with the selective TGF-β receptor ALK4/ALK5 inhibitor EW-7197 resulted in complete inhibition of RAI uptake in CM-treated SMAD-NIS-MSCs, demonstrating the selective stimulation of the SMAD-responsive promoter in SMAD-NIS-MSCs through TGF-β1. Data are represented as means of three independent experiments ± SEMs ($n = 3$; independent samples t test; $^{*}p < 0.05$; $^{**}p < 0.01$; $^{***}p < 0.001$).

secretion at the protein level, tumor cell line-conditioned medium (CM) from each cell line was analyzed by enzyme-linked immunosorbent assay (ELISA) (Figure 1B). All cell lines showed TGF-β1 secretion into the CM, with the highest levels seen in BCPAP-CM.

Stimulation of NIS-mediated RAI accumulation in SMAD-NIS-MSCs by TC cell-CM

In our previous work, SMAD-NIS-MSCs were extensively characterized, and activation of the SMAD-responsive promoter by TGF-β1 was demonstrated: stimulation with TGF-β1 at concentrations ranging from 5 to 25 ng/mL resulted in a 3.4- to 4.9-fold increase in NIS-mediated RAI uptake. Notably, significant activation was observed at concentrations ≥5 ng/mL, with a plateau reached at 20–25 ng/mL.[42]

In the present study, the ability of thyroid tumor cell-derived TGF-β to activate SMAD-mediated NIS expression in SMAD-NIS-MSCs was established.

CM diluted 1:4 with normal medium was used to stimulate SMAD-NIS-MSCs. Stimulation of SMAD-NIS-MSCs with CM from BCPAP, BHT101, and K1 cells resulted in significant stimulation of NIS-mediated iodide accumulation (Figure 1C). To confirm NIS specificity, the NIS-specific inhibitor perchlorate was used in all groups. The results showed complete inhibition of iodide uptake (Figure 1C). To further verify that stimulation of NIS-mediated iodide accumulation in SMAD-NIS-MSCs was mediated by TGF-β1, SMAD-NIS-MSCs stimulated by BCPAP-CM, BHT101-CM, K1-CM, or 10 ng/mL TGF-β1, respectively, were additionally treated with the selective TGF-β receptor ALK4/ALK5 inhibitor EW-7197 (0.5 μmol/L). This resulted in complete inhibition of RAI uptake (Figure 1C). As also shown in our previous studies,[40,42] SMAD-NIS-MSCs cultured with normal medium did not show TGF-β-responsive activation of NIS-mediated iodide accumulation, demonstrating that MSCs secrete low levels of active TGF-β and do not induce significant autocrine activation of the TGF-β signaling pathway.

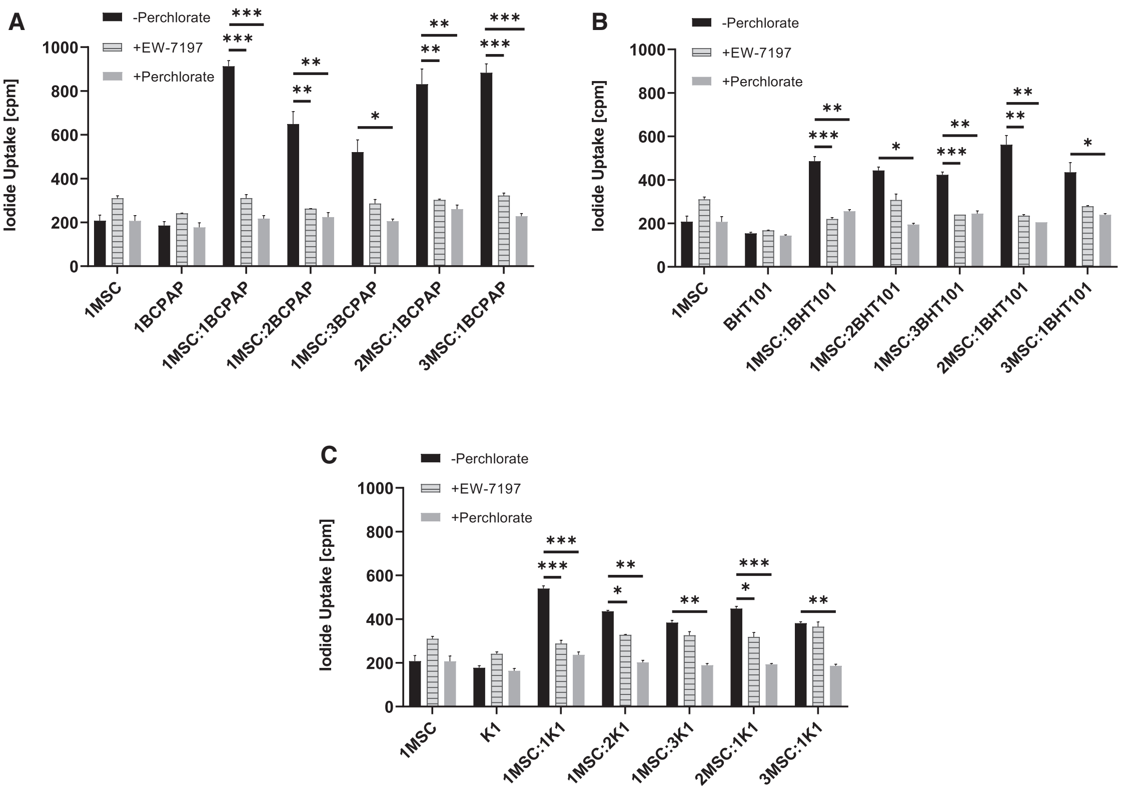

Figure 2. RAI accumulation in SMAD-NIS-MSCs co-cultured with TC cell lines

No RAI uptake was seen in MSCs or tumor cells alone. MSCs co-cultured with BCPAP, BHT101, and K1 cells showed induction of iodide uptake, with the highest levels seen at cell ratios of 1:1 (MSC:BCPAP) (A), 2:1 (MSC:BHT101) (B), and 1:1 (MSC:K1) (C), respectively. Treatment with perchlorate or the TGF-β receptor ALK4/ALK5 inhibitor EW-7197 resulted in inhibition of RAI uptake demonstrating the NIS specificity of iodide accumulation and TGF-β specificity of induction of functional NIS expression, respectively (A–C). Data are represented as means of three independent experiments ± SEMs ($n = 3$; one-way ANOVA; *$p < 0.05$; **$p < 0.01$; ***$p < 0.001$).

Stimulation of NIS-mediated RAI accumulation in SMAD-NIS-MSCs by co-culture with TC cell lines

SMAD-NIS-MSCs were then directly co-cultured with the three tumor cell lines (BCPAP, BHT101, and K1) at different tumor cell-to-MSC ratios. No RAI uptake activity above background levels was seen in SMAD-NIS-MSCs or in tumor cell lines alone, demonstrating the lack of functional NIS expression in these cell lines. SMAD-NIS-MSCs co-cultured with BCPAP showed the highest level of RAI uptake as compared to co-culture with the other two cell lines. The strongest signal was seen at a ratio of 1 MSC:1 BCPAP (Figure 2A). Co-culture with BHT101 also induced NIS expression in SMAD-NIS-MSCs, although at a lower level, with the strongest effect seen at a ratio of 2 MSC:1 BHT101 (Figure 2B). Co-culture with K1 also showed stimulation of NIS expression in SMAD-NIS-MSCs at lower levels, with the strongest effect seen at the ratio 1 MSC:1 K1 (Figure 2C). Treatment with perchlorate confirmed NIS specificity in the RAI uptake assays, and use of the TGF-β receptor ALK4/ALK5 inhibitor EW-7197 demonstrated induction of functional NIS expression in SMAD-NIS-MSCs through TGF-β1 (Figures 2A–2C).

MSCs showed directed migration toward tumor-derived signals

Based on the most consistent *in vitro* data in the BCPAP and K1 cell lines, these two cell lines were then chosen for the following *in vitro* and *in vivo* experiments. The results presented above convincingly demonstrated that the tumor cell lines tested secreted sufficient TGF-β1 to activate the SMAD-responsive promotor in SMAD-NIS-MSCs, thereby driving SMAD-induced functional NIS expression. MSCs have been shown to respond to diverse chemotactic signals by expressing a wide range of functional chemokine receptors.[46] To investigate whether TGF-β1 could also play a role in the directed migration of MSCs, a three-dimensional (3D) chemotaxis assay was applied. SMAD-NIS-MSCs were subjected to untreated normal medium in both chambers to demonstrate a lack of directed migration or chemotaxis and to control for enhanced random migration or chemokinesis (Figure 3A). MSCs subjected to a gradient between untreated normal medium and TGF-β1 (10 ng/mL) showed a non-directed chemotaxis toward recombinant TGF-β1 (Figure 3B) but with an increase in the shift of mean center of mass (yCoM) (Figure 3H) and enhanced mean forward migration index (yFMI) (Figure 3G). MSCs under the influence of a gradient between untreated medium and tumor CM showed

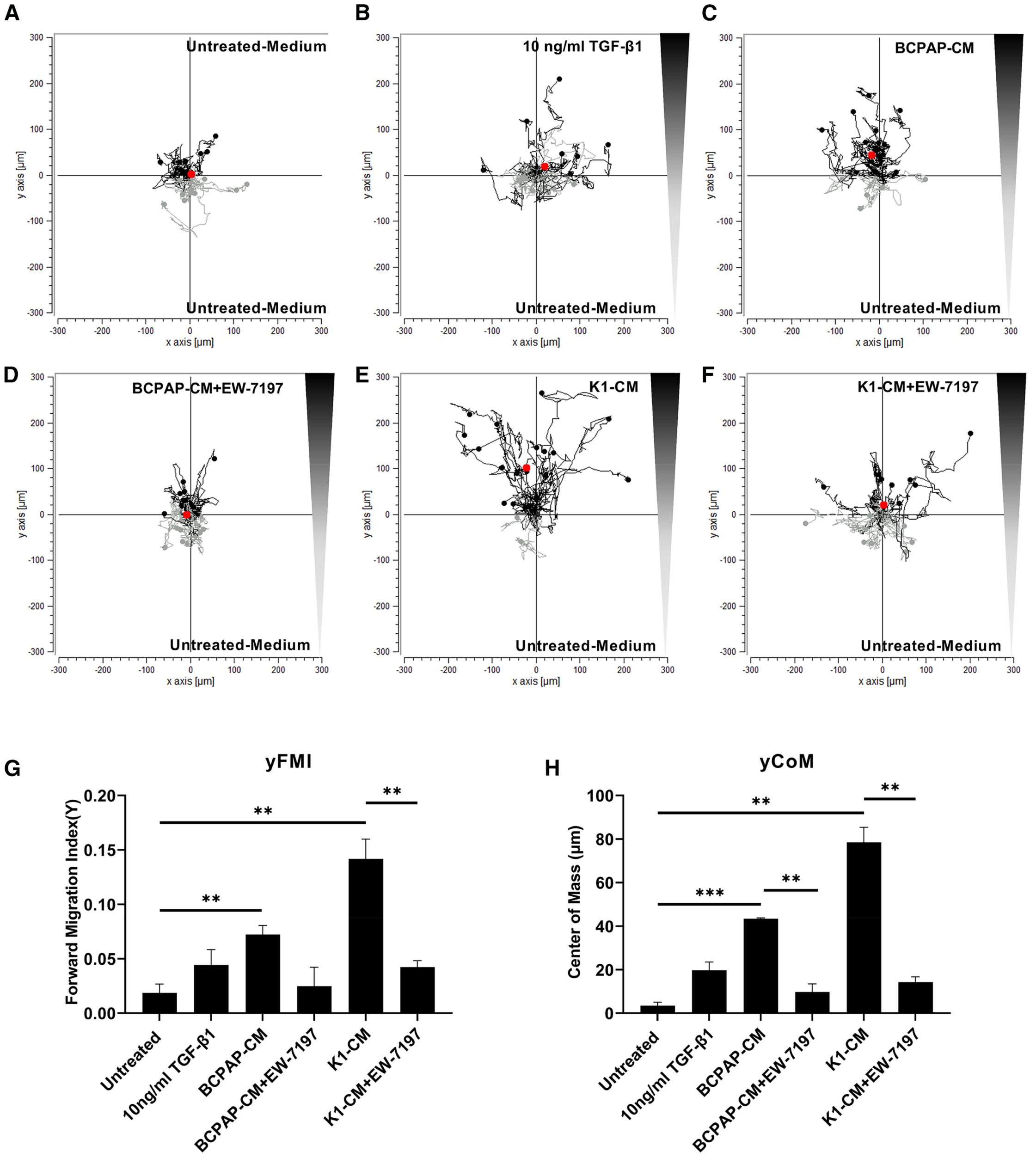

Figure 3. Chemotactic behavior of SMAD-NIS-MSCs evaluated by chemotaxis assay

(A) SMAD-NIS-MSCs subjected to untreated medium in both chambers showed no directed chemotaxis. (B, G, and H) MSCs showed non-directed chemotaxis toward TGF-β1 (B) but with an increase in the shift of mean center of mass (yCoM; red dots) (B and H) and enhanced mean forward migration index (yFMI) (G). (C–H) Cells under the influence of a gradient between untreated medium and tumor CM showed directed chemotaxis (C and E; Rayleigh values $p < 0.05$) and a significant increase in yCoM and yFMI toward the BCPAP-CM and K1-CM compared to untreated medium (G and H; independent samples *t* test; $^{**}p < 0.01$; $^{***}p < 0.001$), which was blocked in the presence of EW-7197 (D and F). Data are represented as mean values ± SEMs from three independent experiments.

directed chemotaxis (Rayleigh values $p < 0.05$) (Figures 3C and 3E) and a significant increase in yCoM and yFMI toward the BCPAP-CM and K1-CM as compared to untreated medium (Figures 3G and 3H) that was blocked in the presence of EW-7197 (Figures 3D, 3F–3H).

In vivo ^{123}I scintigraphy imaging after SMAD-NIS-MSC-mediated NIS gene transfer in TC xenograft mouse models

For *in vivo* studies, BCPAP and K1 cell lines were selected to establish subcutaneous mouse models. After 5–7 weeks, when the subcutaneous tumors had reached a volume of 500–700 mm^3,

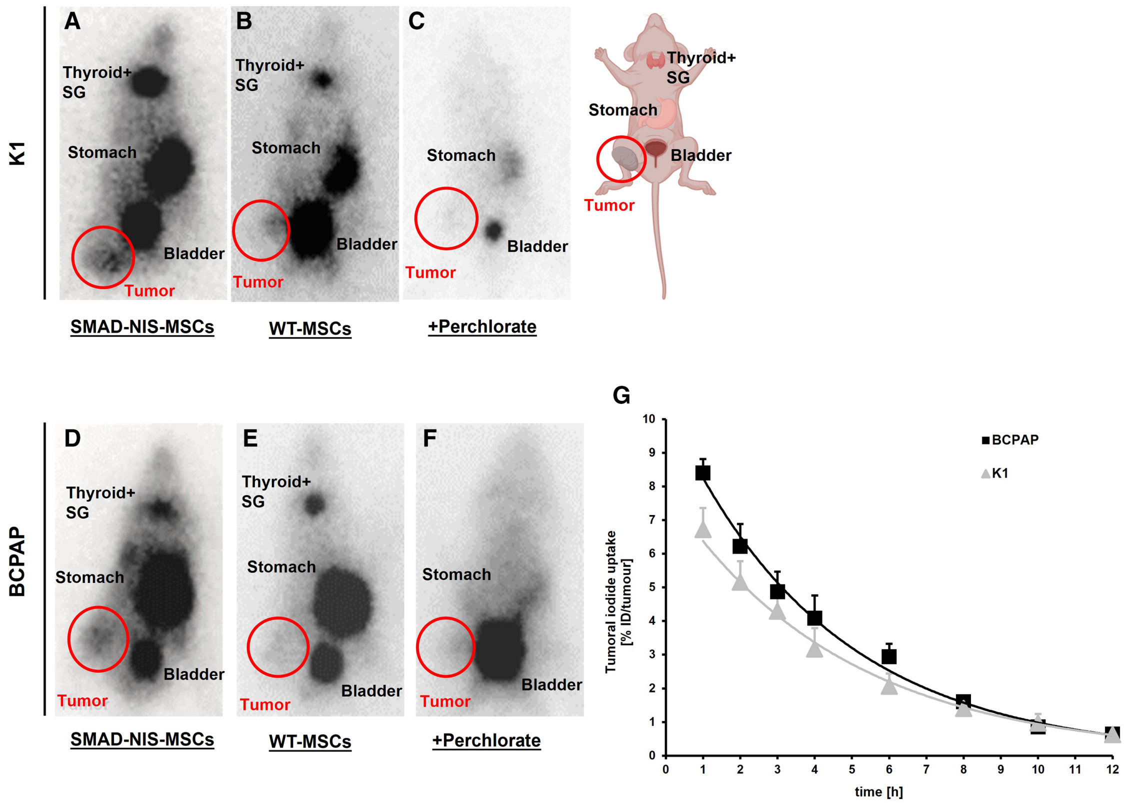

Figure 4. *In vivo* ^{123}I scintigraphy of NIS-mediated iodide uptake in K1 and BCPAP xenografts

(A, C, D, and F) Three i.v. injections of SMAD-NIS-MSCs in mice harboring subcutaneous K1 tumors (A) and BCPAP tumors (D) resulted in tumor-specific RAI uptake that was perchlorate sensitive (C and F). (B and E) Injection of WT-MSCs instead of SMAD-NIS-MSCs resulted in no tumor-specific RAI uptake in either model. (G) RAI retention time in tumors was determined by serial scanning over 12 h. Data are represented as mean values ± SEMs. SG, salivary gland.

5×10^5 SMAD-NIS-MSCs in 500 μL PBS were injected into tumor-bearing nude mice via the tail vein three times in 48-h intervals. Seventy-two hours after the last MSC application, 18.5 MBq ^{123}I was administered intraperitoneally (i.p.), and the RAI biodistribution was analyzed using ^{123}I scintigraphy. The images revealed a maximum tumoral iodide accumulation 1 h post-^{123}I injection with approximately 6.72% ± 0.67% of the injected dose per tumor in K1 subcutaneous tumors after SMAD-NIS-MSC application (Figures 4A and 4G), whereas BCPAP subcutaneous tumors accumulated 8.40% ± 0.42% (Figures 4D and 4G). A biological half-life of 3.7 and 3.9 h and tumor-absorbed doses of 26 and 42 mGy/MBq/g for K1 and BCPAP, respectively, were calculated for ^{131}I. Physiological NIS-mediated RAI uptake was also seen in the thyroid, salivary glands, and stomach. RAI accumulation in the urinary bladder was due to renal elimination of ^{123}I.

To confirm that the tumoral ^{123}I accumulation was NIS mediated, the control group received wild-type MSCs (WT-MSCs) instead of SMAD-NIS-MSCs. These mice showed no RAI uptake in tumors (Figures 4B and 4E). In an additional control group of mice injected with SMAD-NIS-MSCs, pretreatment with perchlorate 30 min prior to ^{123}I administration inhibited the tumoral and physiological accumulation of RAI in both models (Figures 4C and 4F).

NIS and TGF-β1 immunohistochemistry in dissected tumors

After evaluation of functional NIS transgene expression by ^{123}I scintigraphy, NIS immunohistochemical staining was performed on formalin-fixed paraffin-embedded (FFPE) tumor tissue sections to analyze the biodistribution of SMAD-NIS-MSCs within the experimental tumors and in non-target organs (kidney, liver, lung, and spleen). The staining revealed NIS-specific immunoreactivity (red) in both K1 (Figure 5A) and BCPAP xenograft tumors (Figure 5D) throughout the tumor stroma after the application of SMAD-NIS-MSCs, while no NIS-specific immunostaining was observed after WT-MSC injection (Figures 5B and 5E) or in non-target organs (Figures 6A–6H). TGF-β1 immunohistochemistry of subcutaneous BCPAP and K1 tumors demonstrated abundant TGF-β1 expression (brown) within the tumor cells (Figures 5C and 5F).

In vivo ^{131}I therapy studies

Following demonstration of the induction of significant levels of functional NIS expression in BCPAP and K1 xenograft tumors

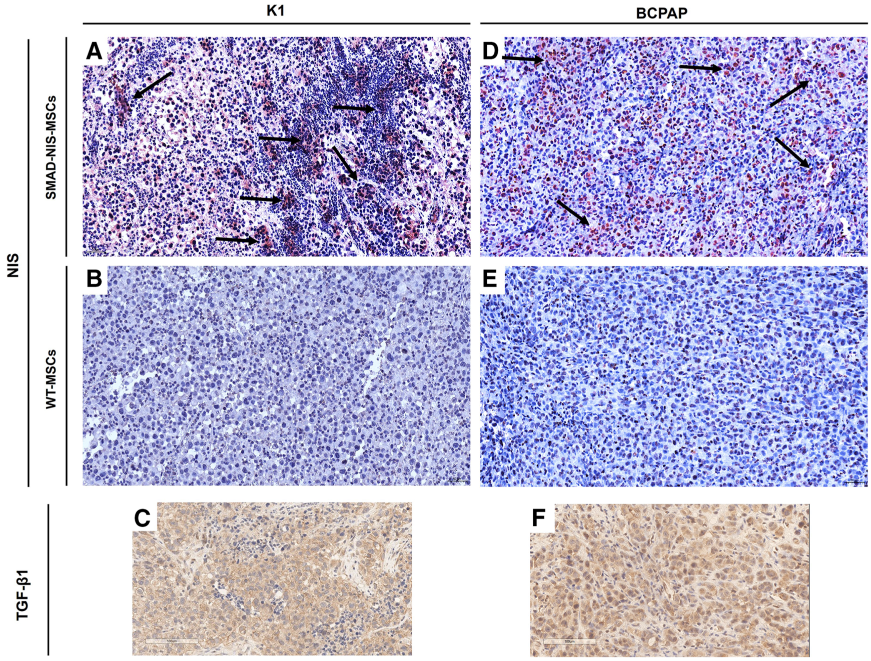

Figure 5. Immunohistochemical analysis of tumoral NIS and TGF-β1 protein expression

(A, B, D, and E) NIS immunohistochemistry confirmed robust NIS protein expression (black arrows) in K1 (A) and BCPAP (D) tumors of mice that were injected systemically with SMAD-NIS-MSCs, while no NIS-specific immunoreactivity was detected after WT-MSC injection (B and E). (C and F) Abundant TGF-β1 protein expression was demonstrated within the K1 (C) and BCPAP (F) tumors. One representative image is shown for each (20× magnification).

after SMAD-NIS-MSC-mediated *NIS* gene transfer by non-invasive ^{123}I scintigraphy imaging confirmed by *ex vivo* NIS immunohistochemistry, a ^{131}I therapy trial was performed in both BCPAP and K1 xenograft mouse models. After three SMAD-NIS-MSC applications in 48-h intervals, animals in the therapy group received a single ^{131}I injection (55.5 MBq) 48 h later. This cycle was repeated 24 h after the first cycle. For the third therapy cycle, a single SMAD-NIS-MSC application was applied, followed by a final ^{131}I injection 48 h later (55.5 MBq) (Figures 7A and 8A).

In mice harboring K1 xenograft tumors, the therapy group (SMAD-NIS-MSCs + ^{131}I) showed a significant delay in tumor growth as compared to control groups receiving WT-MSCs + ^{131}I ($^{**}p < 0.01$), SMAD-NIS-MSCs followed by saline (NaCl) instead of ^{131}I (SMAD-NIS-MSCs + NaCl) ($^{**}p < 0.01$), or NaCl only (NaCl + NaCl) ($^{***}p < 0.001$), all of which exhibited continuous exponential tumor growth (Figure 7B). Furthermore, mice treated with SMAD-NIS-MSCs followed by ^{131}I showed a significantly prolonged median survival of 28 days compared with control groups receiving WT-MSCs + ^{131}I ($^{**}p < 0.01$), SMAD-NIS-MSCs + NaCl ($^{***}p < 0.001$), or NaCl only ($^{***}p < 0.001$), which exhibited significantly shorter median survival times of 18–24 days (Figure 7C).

In mice harboring BCPAP xenografts, treatment with SMAD-NIS-MSCs followed by ^{131}I application also resulted in a significant inhibition of tumor growth as compared to the control groups (WT-MSCs + ^{131}I, $^{*}p < 0.05$; SMAD-NIS-MSCs + NaCl, $^{**}p < 0.01$; NaCl + NaCl, $^{**}p < 0.01$) that showed continuous exponential tumor growth (Figure 8B). This treatment also resulted in significantly prolonged survival when compared to the NaCl-control groups (SMAD-NIS-MSCs + NaCl, median survival 38 days, $^{*}p < 0.05$; NaCl + NaCl, median survival 38 days, $^{**}p < 0.01$). Although not statistically significant, on day 54 after the start of therapy, only 25% of mice that had received WT-MSCs and ^{131}I treatment survived (median survival 42 days), whereas in the therapy group, 67% mice were still alive, demonstrating a robust therapeutic effect (median survival not reached, Figure 8C).

No toxicity was observed in both therapy studies within the observation period.

Ex vivo Ki67 and CD31 analysis in dissected tumors

At the end of the therapy study, the tumors were dissected and stained for the cell proliferation marker Ki67 and a marker for blood vessels (CD31) (Figures S2A and S2D). Both K1 and BCPAP tumors treated with SMAD-NIS-MSCs and ^{131}I (SMAD-NIS-MSCs + ^{131}I) showed a significantly lower proliferation index as compared to the control groups (Figures S2B and S2E). In addition, K1 and BCPAP tumors from the therapy group showed a lower blood vessel density as compared to the control groups (Figures S2C and S2F).

DISCUSSION

Significant progress has been made over the past 2 decades in the development of *NIS* gene therapy. This includes local *NIS* gene delivery as well as systemic approaches for the potential treatment of metastatic disease through the use of oncolytic viruses and non-viral polyplexes.[45] In addition, genetically engineered MSCs have been shown to act as a highly effective, selective and flexible delivery vehicle for *NIS* transgenes in tumor settings.[24,34–44] Genetically engineered MSCs can deliver therapeutic genes deep into critical microenvironments of growing tumors based on their intrinsic tumor homing properties and have been shown to act as an efficient delivery system for systemic *NIS* gene therapy in our recent work.[24,33–44] The mechanisms underlying homing to the tumor are thought to be driven by the inflammatory micromilieu of the tumor and the presence of mediators such as epidermal growth factor (EGF), platelet-derived

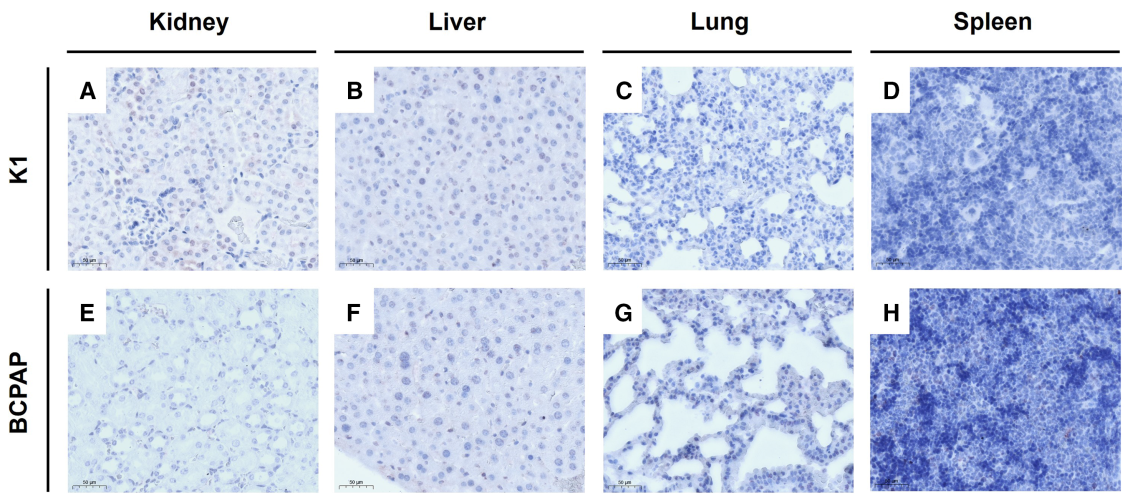

Figure 6. Immunohistochemical analysis of NIS protein expression in non-target organs

NIS immunohistochemistry confirmed no NIS-specific immunostaining in non-target organs of mice harboring K1 (A–D) and BCPAP (E–H) xenograft tumors, following systemic injection of SMAD-NIS-MSCs. One representative image is shown for each (20× magnification).

growth factor, and vascular EGF, as well as TGF-β, among other inflammatory chemokines and growth factors.[23,41] A major focus of our group has been the evaluation of tumor signal-specific promoters to enhance tumor specificity, thereby tailoring the *NIS* gene therapy approach to the individual tumor micromilieu. We have evaluated various tumor-derived signal-specific promoters for MSC-based *NIS* gene therapy approaches,[45] including through the use of the RANTES/CCL5[36,37] and interleukin-6 promoters[34] activated by proinflammatory signals within the tumor micromilieu. In addition, we have demonstrated the use of a synthetic hypoxia-inducible factor-1-responsive promoter that is activated within the hypoxic environments that represents an important feature of most solid tumors.[38]

One of our most promising approaches has been the application of a synthetic TGF-β1-inducible SMAD-responsive promoter. The central role of TGF-β in tumor biology makes the TGF-β/SMAD signaling pathway an attractive option to drive and target NIS expression in the tumor milieu and was first evaluated in a hepatocellular xenograft mouse model.[40,42] Effective recruitment of SMAD-NIS-MSCs to the HuH7 xenografted liver tumor and TGF-β/SMAD-induced NIS expression was demonstrated by ^{123}I scintigraphy and resulted in a significant delay in tumor growth with prolonged survival in ^{131}I-based therapy trials.[42] In the present study, we used SMAD-NIS-MSCs to utilize BRAF^{V600E}-induced stimulation of TGF-β/SMAD signaling in RAI-refractory BRAF^{V600E}-mutant PTC to drive NIS expression, thereby allowing the induction of RAI accumulation in the tumor stroma and destroying tumor cells through the crossfire effect of ^{131}I or other therapeutic radionuclides transported by NIS, such as ^{188}Re or ^{211}At.[36,47–49]

In the present study, functionally relevant TGF-β expression levels were verified in BRAF^{V600E}-mutant PTC and ATC cell lines using quantitative real-time PCR and ELISA. Secretion of TGF-β1 protein into the supernatant as shown by ELISA was confirmed in all three TC cell lines, largely correlating with TGF-β1 mRNA levels. TGF-β1 protein expression was further confirmed in BCPAP and K1 xenograft tumors by immunohistochemical staining. SMAD-NIS-MSCs were confirmed *in vitro* to express significant levels of functional NIS as shown by perchlorate-sensitive, NIS-mediated RAI uptake, upon treatment with CM from the tumor cell lines BCPAP, BHT101, and K1, demonstrating activation of the TGF-β1/SMAD-responsive promoter by tumor-derived signals. Co-incubation with the TGF-β receptor ALK4/ALK5 inhibitor EW-7197 completely inhibited RAI uptake activity in SMAD-NIS-MSCs, confirming that induction of NIS expression was mediated by the TGF-β-inducible SMAD-responsive promoter. In addition, SMAD-NIS-MSCs showed strong induction of NIS-mediated RAI accumulation when co-cultured with the TC cells, with most prominent levels of RAI uptake seen in co-culture with BCPAP (ratio of 1 MSC:1 BCPAP), also completely inhibited by co-incubation with the TGF-β receptor ALK4/ALK5 inhibitor EW-7197.

The *in vitro* chemotaxis assay described here showed the directed chemotaxis of SMAD-NIS-MSCs toward CM derived from BCPAP and K1. This migration could be reduced by the TGF-β receptor ALK4/ALK5 inhibitor EW-7197, further suggesting a role for TGF-β in the directed migration of the MSCs, among other factors. The levels of TGF-β measured in supernatants of TC cell lines BCPAP and K1 do not directly correlate with the effects of CM on the migration of MSCs, underlining that it is a dynamic and complex process involving the response to a wide range of factors, including various chemokines.[46] As outlined above, TGF-β has been established as an important inflammatory mediator for the tumor-selective homing of MSCs.[23,41] This is also supported by our

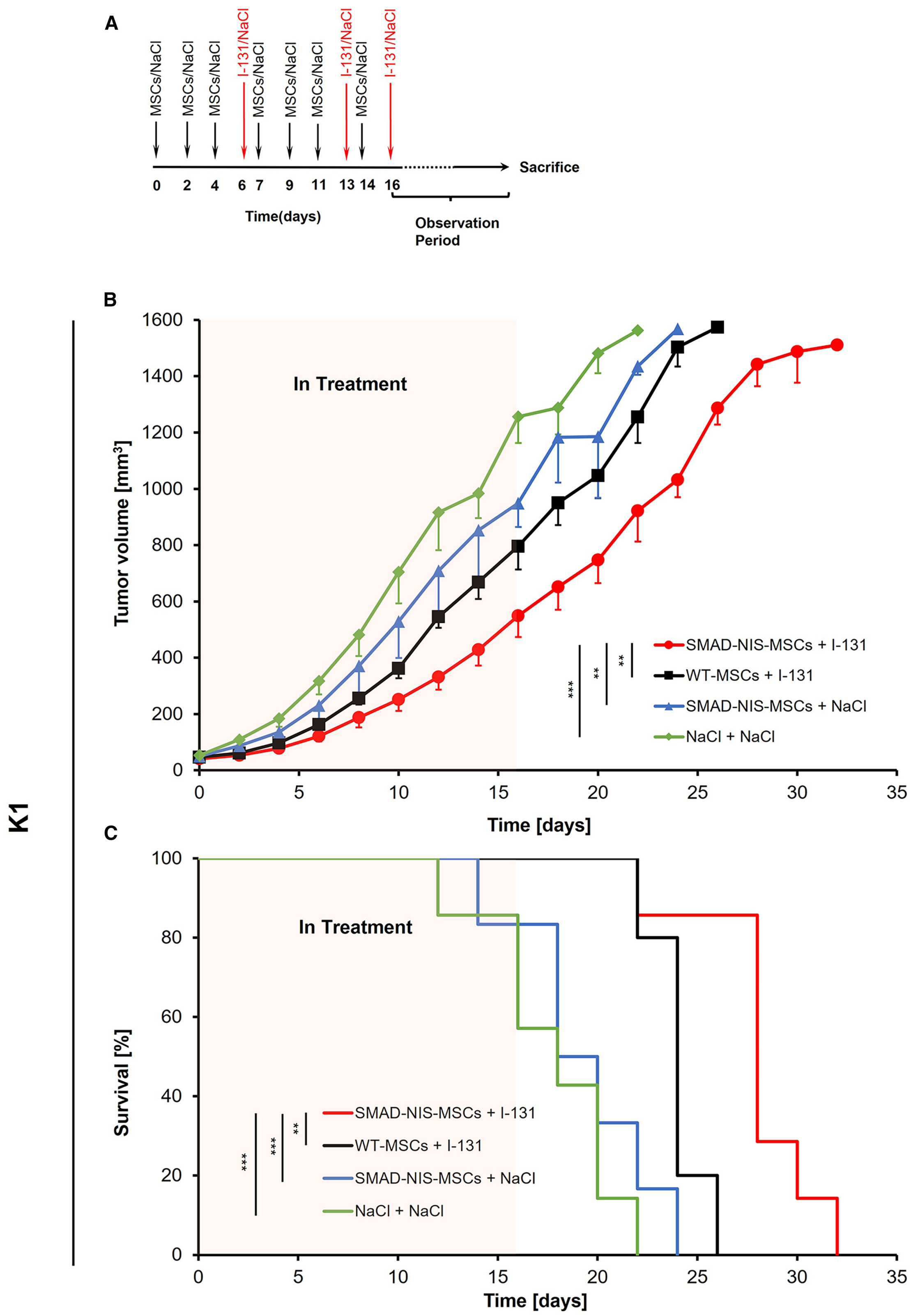

(legend on next page)

findings and suggests a dual role of tumor-derived TGF-β in the context of SMAD-NIS-MSC-driven *NIS* gene therapy in RAI-refractory DTC.

Based on these promising *in vitro* data showing quantitative analysis of TGF-β1 specificity of functional NIS expression induction in SMAD-NIS-MSC using a TGF-β1-inducible SMAD-responsive promoter, the BCPAP and K1 xenograft mouse models were used for *in vivo* ^{123}I scintigraphy imaging studies following application of SMAD-NIS-MSCs. We convincingly demonstrated not only effective recruitment of SMAD-NIS-MSCs into the tumor stroma but also a robust and tumor-selective TGF-β1-driven induction of the *NIS* transgene as shown by tumor-specific, perchlorate-sensitive RAI accumulation in both models. In BCPAP xenograft tumors, higher levels of ^{123}I accumulation were achieved as compared to K1 tumors in line with the higher levels of TGF-β1 expression found in the BCPAP cells.

TGF-β1-driven NIS protein expression in the BCPAP and K1 xenograft tumors of mice treated with SMAD-NIS-MSCs was confirmed by NIS-specific immunoreactivity in the tumor stroma. No NIS expression was found in non-target organs such as liver, lung, spleen, and kidney. These data support our hypothesis that the strategy of employing TGF-β1 to activate NIS transgene expression in the tumor microenvironment results in tumor-selective SMAD promoter-driven functional NIS expression in BRAF^{V600E}-mutant PTC, avoiding the risk of potential side effects in non-target tissues such as the spleen or lung.[23]

The subsequent therapy trial applying SMAD-NIS-MSCs in both BRAF^{V600E}-mutant PTC models followed by therapeutic application of ^{131}I showed significantly reduced tumor growth in both the BCPAP and K1 xenograft mouse tumor models as compared to the control groups. This tumor growth-inhibiting effect also resulted in prolonged survival of the therapy groups. These data were further supported by a significantly reduced proliferation (Ki67) index in the therapy groups and an antiangiogenic effect as shown by reduced blood vessel density (CD31) in the therapy groups as compared to the controls.

Results from our earlier study[40] suggested that the tissue damage induced by the accumulated ^{131}I in the tumor following SMAD-NIS-MSC-mediated *NIS* gene therapy may by itself enhance the inflammatory response in the tumor microenvironment, including stimulation of TGF-β release, thereby leading to a self-energizing therapy cycle with enhanced MSC recruitment and TGF-β-induced SMAD-promoter activation.[40]

In conclusion, our data demonstrate a promising therapy strategy for BRAF^{V600E}-driven RAI-refractory advanced PTC by hijacking the central role of TGF-β/SMAD signaling in these tumors to drive tumor-selective NIS induction using SMAD-NIS-MSC-mediated *NIS* gene delivery, thereby attacking the tumor with its own weapons. As a next crucial step toward clinical translation, this approach needs to be confirmed in clinically more relevant orthotopic and genetically induced PTC mouse models.

MATERIALS AND METHODS

TC cell lines

The human PTC cell line BCPAP (DSMZ catalog no. ACC 273, Braunschweig Germany, RRID: CVCL_0153) and ATC cell line BHT101 (DSMZ catalog no. ACC 279, RRID: CVCL_1085) were cultured in RPMI 1640 culture medium (Sigma-Aldrich, St. Louis, MO) supplemented with 10% (v/v) fetal bovine serum (FBS) (Sigma-Aldrich) and 100 U/mL penicillin/100 μg/mL streptomycin (P/S; Sigma-Aldrich). The human PTC cell line K1 (Sigma-Aldrich catalog no. 92030501, RRID: CVCL_2537) was grown in a 2:1:1 mixture of DMEM high glucose (4.5 g/L, Sigma-Aldrich), Ham's F12 (Sigma-Aldrich), and MCDB 105 (Sigma-Aldrich) supplemented with 10% (v/v) FBS, 2 mM glutamine, and P/S. All cells were maintained at 37°C, 5% CO_2, and 95% relative humidity. Cell culture medium was replaced every 2–3 days, and the cells were passaged at 70%–90% confluency.

As previously described,[50] the TC cell lines BCPAP, K1, and BHT101 harbor the *BRAF*^{V600E} mutation, resulting in the absence of NIS mRNA expression (data not shown).

SMAD-NIS-MSCs

Previously characterized immortalized human bone marrow-derived MSCs[46,51] (positive expression of cluster of differentiation [CD] 29, CD73, CD105, and CD166 and negative for expression of CD34, CD45, CD14, and CD11) were cultured in RPMI 1640 supplemented with 10% (v/v) FBS and P/S. WT-MSCs were stably transfected with a pcDNA6-2ITRNEO-SMAD-NIS expression vector as previously described.[42] Briefly, a synthetic SMAD-responsive promoter, consisting of five tandem repeats of the SMAD-binding element (AGCCAG-ACAGT) and a minimal cytomegalovirus promoter, was cloned upstream of the *NIS* gene. The resulting expression cassette (SMAD promoter-NIS), integrated into the

Figure 7. Therapeutic application of ^{131}I after systemic SMAD-NIS-MSC application in K1 xenograft mouse models

(A) Mice harboring K1 xenograft tumors were treated with three consecutive i.v. SMAD-NIS-MSC injections (in 48 h intervals), followed by an i.p. injection of 55.5 MBq ^{131}I. This cycle was repeated once. For the third cycle, a single SMAD-NIS-MSC was applied, followed by ^{131}I. (B) The treatment with SMAD-NIS-MSCs and ^{131}I (SMAD-NIS-MSC + ^{131}I, $n = 7$) in mice harboring a subcutaneous K1 tumor resulted in a significant delay in tumor growth compared to the control groups receiving WT-MSCs and ^{131}I (WT-MSCs + ^{131}I, $n = 5$, $^{**}p < 0.01$), SMAD-NIS-MSCs and NaCl (SMAD-NIS-MSCs + NaCl, $n = 6$, $^{**}p < 0.01$), or NaCl only (NaCl + NaCl, $n = 7$, $^{***}p < 0.001$). (C) This also led to an increased median survival of 28 days in the therapy group compared to the control groups (WT-MSCs + ^{131}I, $n = 5$, median survival 24 days, $^{**}p < 0.01$; SMAD-NIS-MSCs + NaCl, $n = 6$, median survival 18 days, $^{***}p < 0.001$; NaCl + NaCl, $n = 7$, median survival 18 days, $^{***}p < 0.001$). Data are represented as mean values ± SEMs for tumor growth and percentage for survival plots.

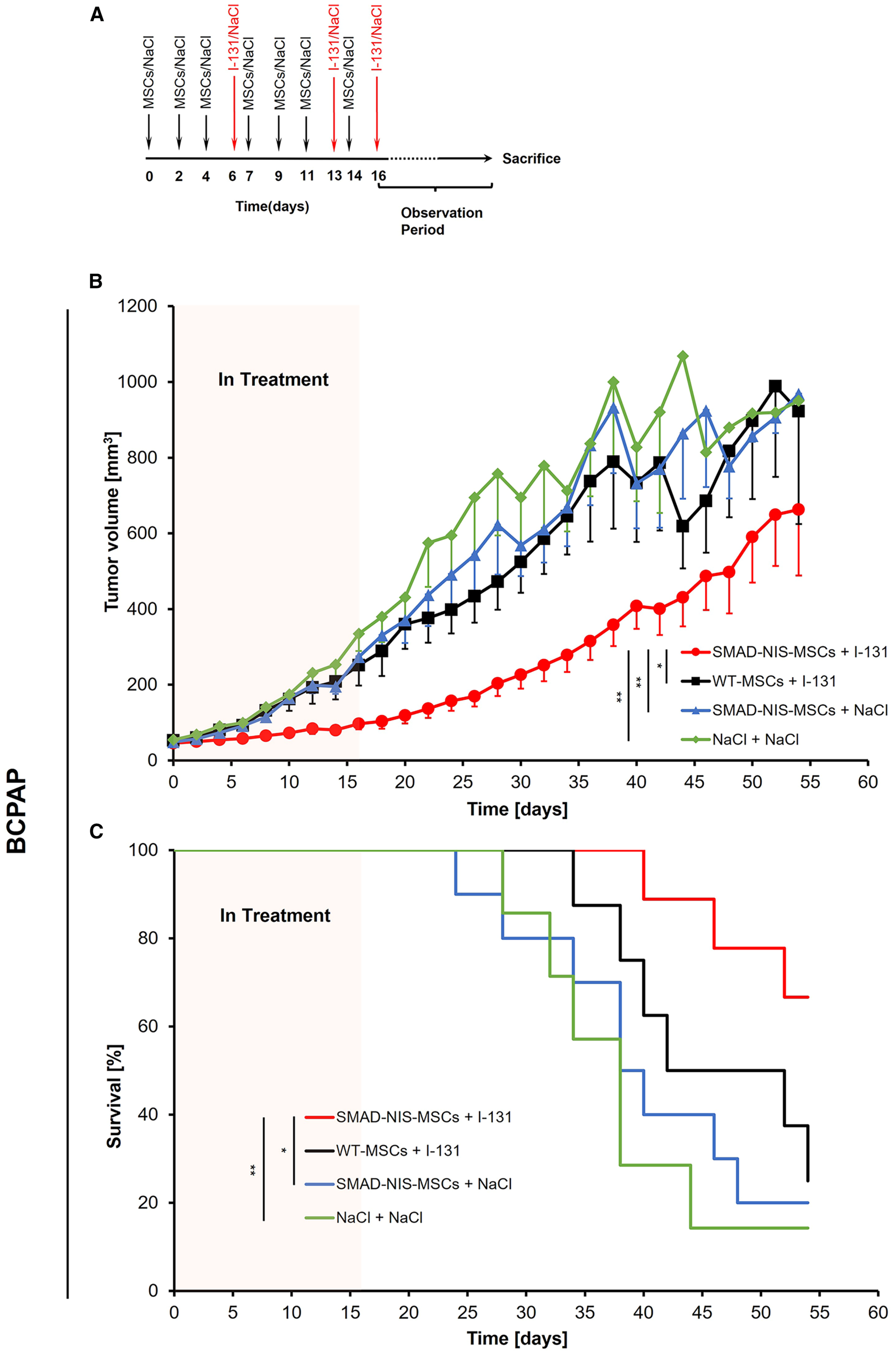

(legend on next page)

pcDNA6-2ITRNEO backbone, was generated using MultiSite Gateway Pro Plus cloning (Thermo Fisher Scientific, Schwerte, Germany). The plasmid construct contains Sleeping Beauty (SB) transposon inverted terminal repeats and a neomycin resistance gene (neo) to facilitate stable genomic integration and selection. The MSCs were then co-electroporated (Neon system) with pcDNA6-2ITRNEO-SMAD-NIS and the SB100X transposase plasmid to enable genomic integration.[52] The stably transfected SMAD-NIS-MSCs were characterized as described previously[42] and were maintained in RPMI 1640 supplemented with 10% (v/v) FBS, P/S, and 0.5 mg/mL G418 (Sigma-Aldrich).

Tumor cell line-CM

We seeded 1×10^6 tumor cells on 100-mm cell culture dishes for 24 h and starved overnight. Medium was exchanged for fresh medium without FBS. Conditioned growth media from the tumor cell lines were collected after 48 h, centrifuged, and stored at −80°C.

Quantitative real-time PCR

Total RNA from tumor cells was isolated with TRIzol Reagent (Invitrogen, Carlsbad, CA) according to the manufacturer's instructions. Reverse transcription was performed using SuperScript III reverse transcriptase (Invitrogen). Quantitative real-time-PCR was done on a Lightcycler 96 System (Roche Diagnostics, Mannheim, Germany) using the SYBR Green PCR master mix. The following primers were used: *ACTB* (β-actin): forward primer (5′-AGAAAA TCTGGCACCACACC-3′), reverse primer (5′-TAGCACAGCCTG GATAGCAA-3′); *R18s* (18s rRNA): forward primer (5′- CAGCC ACCCGAGATTGAGCA-3′), reverse primer (5′- TAGTAGCGAC GGGCGGTGTG-3′); *TGFB1*: forward primer (5′-CAGCACGTGG AGCTGTACC-3′), reverse primer (5′-AAGATAACCACTCTGGC GAGTC-3′); *TGFB2*: forward primer (5′-GTGCTTTGGATGCGGC CTA-3′), reverse primer (5′-GGCATGCTCCAGCACAGAA-3′); *TG FB3*: forward primer (5′-CAAAGGCGTGGACAATGAGG-3′), reverse primer (5′-ACTTCCAGCCCAGATCCTGT-3′); *NIS*: forward primer (5′-TGCTAAGTGGCTTCTGGGTTGT-3′), reverse primer (5′-ATG CTGGTGGATGCTGTGCTGA-3′). Relative expression levels of *TGFB1*, *TGFB2*, *TGFB3*, and *NIS* were calculated from ΔΔCt values normalized to internal *ACTB* and *R18s*.

ELISA

An ELISA assay for TGF-β1 was performed on CM using the human TGF-β1 DuoSet ELISA kit (R&D Systems, Abingdon, UK) following the manufacturer's instructions.

CM stimulation of the SMAD-NIS-MSCs and ^{125}I uptake assay

We seeded 0.3×10^6 SMAD-NIS-MSCs on 6-well plates. After starvation with serum-free medium overnight, medium was changed to 80% (v/v) normal culture medium and 20% (v/v) tumor CM for 24 h. NIS-mediated ^{125}I uptake was determined at steady-state conditions as described previously.[53] For control experiments validating the inducibility of the SMAD-based promoter, the TGF-β receptor ALK4/ALK5 inhibitor EW-7197 (0.5 μmol/L vactosertib; SelleckChem, Houston, TX) was used.[40] The NIS-specific inhibitor perchlorate ($KClO_4$; 100 mM; Merck Millipore, Burlington, MA) was added to verify NIS specificity of uptake. Results were normalized to cell viability and are shown as counts per minute/A620.

Cell viability assay

The commercially available MTT (3-(4,5-dimethylthiazol-2-yl)-2,5-diphenyltetrazolium bromide) assay (Sigma-Aldrich) was performed according to the manufacturer's recommendations. The absorbance of the formazan product was measured on a Sunrise Microplate Absorbance Reader (Tecan, Männedorf, Switzerland) at a wavelength of 620 nm using the software Magellan (Tecan).

Co-culture of SMAD-NIS-MSCs and tumor cell lines

TGF-β-induced functional NIS expression in SMAD-NIS-MSCs was also measured by ^{125}I uptake assay in SMAD-NIS-MSCs that were co-cultured with the TC cell lines at different ratios. We defined 1.5×10^5 of tumor cells and 1.5×10^5 MSCs as a 1:1 ratio. To confirm that the activation of the SMAD-responsive promoter in SMAD-NIS-MSCs was TGF-β induced, co-incubations with the TGF-β receptor ALK4/ALK5 inhibitor EW-7197 (0.5 μmol/L) were performed.

3D migration assay

Migration assays were performed using the μ-slide Chemotaxis 3D system (ibidi, Martinsried, Germany). SMAD-NIS-MSCs were seeded in collagen I (0.5×10^6) and exposed to a gradient between serum-free medium and BCPAP-CM or K1-CM with or without the TGF-β receptor ALK4/ALK5 inhibitor EW-7197. Monitoring of the migration assay was done as previously described.[54] Twenty randomly selected cells were manually tracked and analyzed with Chemotaxis and Migration Tool software (ibidi, RRID: SCR_022708). The FMI and the CoM were calculated using the ibidi Chemotaxis and Migration Tool for all tracked cells to quantify the migratory behavior of the SMAD-NIS-MSCs. As a key parameter for evaluating directed chemotactic cell migration, the FMI quantifies the efficiency of forward movement in response to a chemotactic gradient. The CoM represents the average position of all

Figure 8. Therapeutic application of ^{131}I after systemic SMAD-NIS-MSC application in BCPAP xenograft mouse models

Mice harboring BCPAP xenograft tumors were treated with three consecutive i.v. SMAD-NIS-MSC injections (in 48 h intervals), followed by an i.p. injection of 55.5 MBq ^{131}I. This cycle was repeated once. For the third cycle, a single SMAD-NIS-MSC was applied followed by ^{131}I (A). As for therapy of mice harboring BCPAP tumors (SMAD-NIS-MSCs + ^{131}I, n = 9), tumor growth was significantly reduced as compared to the controls (WT-MSCs + ^{131}I, n = 8, *$p < 0.05$; SMAD-NIS-MSCs + NaCl, n = 10, **$p < 0.01$; NaCl + NaCl, n = 7, **$p < 0.01$) (B), with prolongation of survival time when compared to the NaCl-control groups (SMAD-NIS-MSCs + NaCl, n = 10, median survival 38 days, *$p < 0.05$; NaCl + NaCl, n = 7, median survival 38 days, **$p < 0.01$). Although not statistically significant, a robust increase in survival was also observed compared with the WT-MSCs + ^{131}I group, where on day 54 after the start of therapy only 25% of mice survived, whereas in the therapy group 67% mice were still alive (C). Data are represented as mean values ± SEMs for tumor growth and percentage for survival plots.

individual cell endpoints. The Rayleigh test was used to evaluate the uniformity of circular distribution of cell endpoints.

Animals

Five-week-old female CD1 nu/nu mice (Charles River, Sulzfeld, Germany, RRID: IMSR_CRL:086) were maintained under specific pathogen-free conditions. Mice had access to mouse chow and water *ad libitum*. The experiments were authorized by the regional governmental commission for animals of Upper Bavaria (Regierung von Oberbayern).

K1 and BCPAP xenograft tumors

To establish xenograft tumors, 2×10^6 K1 cells or 15×10^6 BCPAP cells in 50 and 100 μL PBS, respectively, and Matrigel (1:1, BD Biosciences, Heidelberg, Germany) were injected subcutaneously into the right flank region of mice as described previously.[24] Tumor volumes were regularly measured using a caliper and calculated using the equation: length × width × height × 0.52. Mice were sacrificed when the tumor exceeded a volume of 1,500 mm^3.

Pyrosequencing of xenograft tumors

The presence of $BRAF^{V600E}$ mutation was confirmed in K1 and BCPAP xenograft tumors by pyrosequencing as described previously,[55] using the PyroMark Gold Q24 kit (Qiagen, Hilden, Germany). Data were subsequently analyzed using the PyroMark Q24 software (Qiagen, RRID: SCR_018620).

^{123}I scintigraphy imaging study

When subcutaneous tumors (K1 and BCPAP) reached a volume of approximately 500 mm^3, drinking water was supplemented with 5 mg/mL L-thyroxine (L-T4; Sigma-Aldrich). In addition, the animals were given an iodine-deficient diet (ssniff Spezialdiäten GmbH, Soest, Germany) for 10 days prior to RAI application to minimize iodine uptake by the thyroid gland and thus enhance tumoral iodine accumulation. We applied 5×10^5 SMAD-NIS-MSCs or WT-MSCs in PBS three times every second day via the tail vein. Seventy-two hours after the last MSC injection, 18.5 MBq (0.5 mCi) of ^{123}I (GE Healthcare, Braunschweig, Germany) were injected i.p. Each mouse in the control group received 2 mg of the competitive NIS inhibitor sodium perchlorate (Sigma-Aldrich) administered i.p. 30 min before ^{123}I injection. RAI biodistribution was measured using a gamma camera (e.cam; Siemens, Erlangen, Germany) equipped with a low-energy, high-resolution collimator. To evaluate the tumoral RAI uptake, regions of interest were selected and the percentage of the total injected RAI dose per tumor (% ID/tumor) was calculated using the software HERMES GOLD (Hermes Medical Solutions, Stockholm, Sweden). Calculations for the dosimetry of ^{131}I were done according to the medical internal radiation dose technique with a radiation dose assessment resource dose factor as previously described.[43]

NIS and TGF-β1 immunohistochemistry

K1 and BCPAP xenograft tumors and other organs (kidney, liver, lung, and spleen) were dissected from all the mice after the imaging study and embedded in paraffin. Immunohistochemical staining of NIS (antibody MAB3564, clone FP5A, Merck Millipore; 1:500 dilution, RRID: AB_2189816)[56] and TGF-β1 (antibody ab92486, polyclonal, Abcam, Cambridge, UK; 1:500 dilution, RRID: AB_10562492)[42] was performed on FFPE tumor sections as described previously.

^{131}I therapy study

Therapy trials were started when the tumors (K1 and BCPAP) reached an average size of 50 mm^3. Ten days before the RAI injection, all mice received drinking water supplemented with 5 mg/mL L-T4 and an iodine-deficient diet as described above. For the therapy group, SMAD-NIS-MSCs (5×10^5 cells/500 μL PBS) were injected intravenously (i.v.) three times on every second day followed by 55.5 MBq ^{131}I (GE Healthcare) application 48 h after the last SMAD-NIS-MSC injection (K1: SMAD-NIS-MSCs + ^{131}I, $n = 7$; BCPAP: SMAD-NIS-MSCs + ^{131}I, $n = 9$). Twenty-four hours after the RAI application, the cycle was repeated. Therapy was completed by one additional MSC injection followed by a third ^{131}I application (Figures 6A and 7A). In the control groups, mice were injected with WT-MSCs instead of SMAD-NIS-MSCs (K1: WT-MSCs + ^{131}I, $n = 5$; BCPAP: WT-MSCs + ^{131}I, $n = 8$). As further controls, a subset of mice received NaCl instead of ^{131}I (K1: SMAD-NIS-MSCs + NaCl, $n = 6$; BCPAP: SMAD-NIS-MSCs + NaCl, $n = 10$), and another group was injected with NaCl only (K1: NaCl + NaCl, $n = 7$; BCPAP: NaCl + NaCl, $n = 7$). Tumor volume was evaluated every 2–3 days, and mice were sacrificed when the tumor volume exceeded 1,500 mm^3 or when tumors started to ulcerate.

Ex vivo immunofluorescence staining

Frozen tissue sections of dissected tumors from the ^{131}I therapy study were used for indirect immunofluorescence staining of Ki67 (antibody ab16667, clone SP6, Abcam; 1:1,000 dilution, RRID: AB_302459) (cellular proliferation) and CD31 (antibody 550274, clone MEC 13.3, BD Pharmingen, Heidelberg, Germany; 1:200 dilution, RRID: AB_393571) (blood vessel density) as described previously.[57] Stained sections were scanned with the Pannoramic MIDI digital slide scanner with identical exposure time. Quantification of the percentage of the cells positive for Ki67 and areas positive for CD31 in the tumors was performed by evaluation of five high-power fields per tumor using ImageJ software (NIH, Bethesda, MD; RRID: SCR_003070). Results are presented as means ± standard errors of the mean (SEMs).

Statistical analysis

All *in vitro* experiments were repeated at least in triplicate. Results are presented as means ± SEMs, mean fold change ± SEM, or percentage for survival plots and analyzed using SPSS 26 (IBM SPSS Statistics, Armonk, NY; RRID: SCR_002865). Statistical significance was tested by independent samples *t* test and one-way ANOVA (data with homogeneous variances) or Welch test (data with unequal variances) for comparison of more than two groups. For the post hoc test, the Tukey-honestly significant difference test was used for data with homogeneous variances, and Tamhane's T2 test was used for

data with unequal variances. For *in vivo* experiments, the one-way ANOVA or Welch test was performed for tumor volumes, and the log rank test was used for the survival plots. p values < 0.05 were considered statistically significant (*p < 0.05; **p < 0.01; ***p < 0.001).

DATA AND CODE AVAILABILITY

The data generated in this study are available upon request from the corresponding author.

ACKNOWLEDGMENTS

This work was supported by the Deutsche Forschungsgemeinschaft within the Collaborative Research Center SFB 824 (project C8) (to C.S.), Wilhelm Sander-Stiftung (grant no. 2021.094.1 to C.S.), Deutsche Krebshilfe (grant no. 70114896 to C.S.), Förderprogramm für Forschung und Lehre (FöFoLe) (Reg. no. 1031 to V.F.K.) of the medical faculty of LMU Munich, and the Munich Clinician Scientist Program Track FöFoLe+ (Reg. no. 044 to V.F.K.) of the medical faculty of the LMU Munich. We are grateful to Dr. Barbara von Ungern-Sternberg, Rosel Oos, Giovanna Palumbo, and Dr. Markus Strigl (Department of Nuclear Medicine, University Hospital of Munich, LMU Munich) for their valuable help in performing the imaging and animal studies. We thank Anke Fischer and Alexandra Wechselberger (Nelson lab, University Hospital of Munich, LMU Munich) for the setup and support with the migration assay. We are also thankful to Olga Seelbach and the Comparative Experimental Pathology (CEP) team (Institute of Pathology, School of Medicine, TUM University Hospital, Klinikum Rechts der Isar, Technical University of Munich) for the immunohistochemical staining, as well as to Prof. Doris Mayr and Marco Henrichs (Institute of Pathology, LMU Munich) for preparation of the paraffin-embedded slides. Furthermore, we thank Prof. Dr. Julia Mayerle, Dr. Ivonne Regel, and Dr. Ujjwal Mahajan (Department of Internal Medicine II, University Hospital of Munich, LMU Munich) for sharing their lab equipment.

AUTHOR CONTRIBUTIONS

Y.H., conceptualization, formal analysis, investigation, visualization, methodology, and writing – original draft. V.F.K., formal analysis, investigation, visualization, methodology, and writing – original draft. J.N., writing – review & editing. K.A.S., conceptualization, investigation, and writing – review and editing. C. Stauss, investigation. N.S., investigation and writing – review & editing. R.S., investigation. C.K., investigation and writing – review & editing. J.K., investigation, methodology, and writing – review & editing. C.Z., formal analysis. K.S., investigation, methodology, and writing – review & editing. J.C.M., conceptualization and writing – review & editing. W.A.W., conceptualization and writing – review & editing. P.B., conceptualization and resources. S.I.Z., resources and writing – review & editing. P.J.N., conceptualization, supervision, funding acquisition, validation, methodology, and writing – review & editing. C. Spitzweg, conceptualization, resources, supervision, funding acquisition, validation, methodology, project administration, and writing – original draft and review & editing.

DECLARATION OF INTERESTS

The authors declare no competing interests.

SUPPLEMENTAL INFORMATION

Supplemental information can be found online at https://doi.org/10.1016/j.ymthe.2025.10.033.

REFERENCES

1. Haugen, B.R., Alexander, E.K., Bible, K.C., Doherty, G.M., Mandel, S.J., Nikiforov, Y.E., Pacini, F., Randolph, G.W., Sawka, A.M., Schlumberger, M., et al. (2016). 2015 American Thyroid Association Management Guidelines for Adult Patients with Thyroid Nodules and Differentiated Thyroid Cancer: The American Thyroid Association Guidelines Task Force on Thyroid Nodules and Differentiated Thyroid Cancer. Thyroid *26*, 1–133. https://doi.org/10.1089/thy.2015.0020.
2. Spitzweg, C., Bible, K.C., Hofbauer, L.C., and Morris, J.C. (2014). Advanced radioiodine-refractory differentiated thyroid cancer: the sodium iodide symporter and other emerging therapeutic targets. Lancet Diabetes Endocrinol. *2*, 830–842. https://doi.org/10.1016/s2213-8587(14)70051-8.
3. Portulano, C., Paroder-Belenitsky, M., and Carrasco, N. (2014). The Na+/I- symporter (NIS): mechanism and medical impact. Endocr. Rev. *35*, 106–149. https://doi.org/10.1210/er.2012-1036.
4. Spitzweg, C., Harrington, K.J., Pinke, L.A., Vile, R.G., and Morris, J.C. (2001). Clinical review 132: The sodium iodide symporter and its potential role in cancer therapy. J. Clin. Endocrinol. Metab. *86*, 3327–3335. https://doi.org/10.1210/jcem.86.7.7641.
5. Riesco-Eizaguirre, G., Gutiérrez-Martínez, P., García-Cabezas, M.A., Nistal, M., and Santisteban, P. (2006). The oncogene BRAF V600E is associated with a high risk of recurrence and less differentiated papillary thyroid carcinoma due to the impairment of Na+/I- targeting to the membrane. Endocr. Relat. Cancer *13*, 257–269. https://doi.org/10.1677/erc.1.01119.
6. Fagin, J.A., and Wells, S.A., Jr. (2016). Biologic and Clinical Perspectives on Thyroid Cancer. N. Engl. J. Med. *375*, 1054–1067. https://doi.org/10.1056/NEJMra1501993.
7. Pozdeyev, N., Gay, L.M., Sokol, E.S., Hartmaier, R., Deaver, K.E., Davis, S., French, J. D., Borre, P.V., LaBarbera, D.V., Tan, A.C., et al. (2018). Genetic Analysis of 779 Advanced Differentiated and Anaplastic Thyroid Cancers. Clin. Cancer Res. *24*, 3059–3068. https://doi.org/10.1158/1078-0432.CCR-18-0373.
8. Nikiforova, M.N., Kimura, E.T., Gandhi, M., Biddinger, P.W., Knauf, J.A., Basolo, F., Zhu, Z., Giannini, R., Salvatore, G., Fusco, A., et al. (2003). BRAF mutations in thyroid tumors are restricted to papillary carcinomas and anaplastic or poorly differentiated carcinomas arising from papillary carcinomas. J. Clin. Endocrinol. Metab. *88*, 5399–5404. https://doi.org/10.1210/jc.2003-030838.
9. Cancer Genome Atlas Research Network (2014). Integrated genomic characterization of papillary thyroid carcinoma. Cell *159*, 676–690. https://doi.org/10.1016/j.cell.2014.09.050.
10. Riesco-Eizaguirre, G., Rodríguez, I., De la Vieja, A., Costamagna, E., Carrasco, N., Nistal, M., and Santisteban, P. (2009). The BRAFV600E oncogene induces transforming growth factor beta secretion leading to sodium iodide symporter repression and increased malignancy in thyroid cancer. Cancer Res. *69*, 8317–8325. https://doi.org/10.1158/0008-5472.CAN-09-1248.
11. Costamagna, E., García, B., and Santisteban, P. (2004). The functional interaction between the paired domain transcription factor Pax8 and Smad3 is involved in transforming growth factor-beta repression of the sodium/iodide symporter gene. J. Biol. Chem. *279*, 3439–3446. https://doi.org/10.1074/jbc.M307138200.
12. Matoba, H., Sugano, S., Yamaguchi, N., and Miyachi, Y. (1998). Expression of transforming growth factor-β1 and transforming growth factor-β type-II receptor mRNA in papillary thyroid carcinoma. Horm. Metab. Res. *30*, 624–628. https://doi.org/10.1055/s-2007-978946.
13. Kimura, E.T., Kopp, P., Zbaeren, J., Asmis, L.M., Ruchti, C., Maciel, R.M., and Studer, H. (1999). Expression of transforming growth factor beta1, beta2, and beta3 in multinodular goiters and differentiated thyroid carcinomas: a comparative study. Thyroid *9*, 119–125. https://doi.org/10.1089/thy.1999.9.119.
14. Mincione, G., Tarantelli, C., Vianale, G., Di Marcantonio, M.C., Cotellese, R., Francomano, F., Di Nicola, M., Costantini, E., Cichella, A., and Muraro, R. (2014). Mutual regulation of TGF-beta1, TbetaRII and ErbB receptors expression in human thyroid carcinomas. Exp. Cell Res. *327*, 24–36. https://doi.org/10.1016/j.yexcr.2014.06.012.
15. Zhang, J., Wang, Y., Li, D., and Jing, S. (2014). Notch and TGF-beta/Smad3 pathways are involved in the interaction between cancer cells and cancer-associated fibroblasts in papillary thyroid carcinoma. Tumour Biol. *35*, 379–385. https://doi.org/10.1007/s13277-013-1053-z.
16. Massague, J. (1998). TGF-beta signal transduction. Annu. Rev. Biochem. *67*, 753–791. https://doi.org/10.1146/annurev.biochem.67.1.753.
17. Ahmadi, A., Najafi, M., Farhood, B., and Mortezaee, K. (2019). Transforming growth factor-beta signaling: Tumorigenesis and targeting for cancer therapy. J. Cell. Physiol. *234*, 12173–12187. https://doi.org/10.1002/jcp.27955.
18. Colletta, G., Cirafici, A.M., and Di Carlo, A. (1989). Dual effect of transforming growth factor beta on rat thyroid cells: inhibition of thyrotropin-induced proliferation and reduction of thyroid-specific differentiation markers. Cancer Res. *49*, 3457–3462.
19. Kawaguchi, A., Ikeda, M., Endo, T., Kogai, T., Miyazaki, A., and Onaya, T. (1997). Transforming growth factor-beta1 suppresses thyrotropin-induced Na+/I- symporter messenger RNA and protein levels in FRTL-5 rat thyroid cells. Thyroid *7*, 789–794. https://doi.org/10.1089/thy.1997.7.789.

20. Knauf, J.A., Sartor, M.A., Medvedovic, M., Lundsmith, E., Ryder, M., Salzano, M., Nikiforov, Y.E., Giordano, T.J., Ghossein, R.A., and Fagin, J.A. (2011). Progression of BRAF-induced thyroid cancer is associated with epithelial-mesenchymal transition requiring concomitant MAP kinase and TGFbeta signaling. Oncogene *30*, 3153–3162. https://doi.org/10.1038/onc.2011.44.

21. Luckett, K.A., Cracchiolo, J.R., Krishnamoorthy, G.P., Leandro-Garcia, L.J., Nagarajah, J., Saqcena, M., Lester, R., Im, S.Y., Zhao, Z., Lowe, S.W., et al. (2021). Co-inhibition of SMAD and MAPK signaling enhances 124I uptake in BRAF-mutant thyroid cancers. Endocr. Relat. Cancer *28*, 391–402. https://doi.org/10.1530/ERC-21-0017.

22. Dvorak, H.F. (1986). Tumors: wounds that do not heal. Similarities between tumor stroma generation and wound healing. N. Engl. J. Med. *315*, 1650–1659. https://doi.org/10.1056/NEJM198612253152606.

23. Hagenhoff, A., Bruns, C.J., Zhao, Y., von Lüttichau, I., Niess, H., Spitzweg, C., and Nelson, P.J. (2016). Harnessing mesenchymal stem cell homing as an anticancer therapy. Expert Opin. Biol. Ther. *16*, 1079–1092. https://doi.org/10.1080/14712598.2016.1196179.

24. Knoop, K., Kolokythas, M., Klutz, K., Willhauck, M.J., Wunderlich, N., Draganovici, D., Zach, C., Gildehaus, F.J., Böning, G., Göke, B., et al. (2011). Image-guided, tumor stroma-targeted 131I therapy of hepatocellular cancer after systemic mesenchymal stem cell-mediated NIS gene delivery. Mol. Ther. *19*, 1704–1713. https://doi.org/10.1038/mt.2011.93.

25. Niess, H., Bao, Q., Conrad, C., Zischek, C., Notohamiprodjo, M., Schwab, F., Schwarz, B., Huss, R., Jauch, K.W., Nelson, P.J., and Bruns, C.J. (2011). Selective targeting of genetically engineered mesenchymal stem cells to tumor stroma microenvironments using tissue-specific suicide gene expression suppresses growth of hepatocellular carcinoma. Ann. Surg. *254*, 767–774, discussion: 774–775. https://doi.org/10.1097/SLA.0b013e3182368c4f.

26. Conrad, C., Hüsemann, Y., Niess, H., von Luettichau, I., Huss, R., Bauer, C., Jauch, K.W., Klein, C.A., Bruns, C., and Nelson, P.J. (2011). Linking transgene expression of engineered mesenchymal stem cells and angiopoietin-1-induced differentiation to target cancer angiogenesis. Ann. Surg. *253*, 566–571. https://doi.org/10.1097/SLA.0b013e3181fcb5d8.

27. Zischek, C., Niess, H., Ischenko, I., Conrad, C., Huss, R., Jauch, K.W., Nelson, P.J., and Bruns, C. (2009). Targeting tumor stroma using engineered mesenchymal stem cells reduces the growth of pancreatic carcinoma. Ann. Surg. *250*, 747–753. https://doi.org/10.1097/SLA.0b013e3181bd62d0.

28. Conrad, C., Gupta, R., Mohan, H., Niess, H., Bruns, C.J., Kopp, R., von Luettichau, I., Guba, M., Heeschen, C., Jauch, K.W., et al. (2007). Genetically engineered stem cells for therapeutic gene delivery. Curr. Gene Ther. *7*, 249–260. https://doi.org/10.2174/156652307781369119.

29. von Einem, J.C., Guenther, C., Volk, H.D., Grütz, G., Hirsch, D., Salat, C., Stoetzer, O., Nelson, P.J., Michl, M., Modest, D.P., et al. (2019). Treatment of advanced gastrointestinal cancer with genetically modified autologous mesenchymal stem cells: Results from the phase 1/2 TREAT-ME-1 trial. Int. J. Cancer *145*, 1538–1546. https://doi.org/10.1002/ijc.32230.

30. von Einem, J.C., Peter, S., Günther, C., Volk, H.D., Grütz, G., Salat, C., Stoetzer, O., Nelson, P.J., Michl, M., Modest, D.P., et al. (2017). Treatment of advanced gastrointestinal cancer with genetically modified autologous mesenchymal stem cells - TREAT-ME-1 - a phase I, first in human, first in class trial. Oncotarget *8*, 80156–80166. https://doi.org/10.18632/oncotarget.20964.

31. Niess, H., von Einem, J.C., Thomas, M.N., Michl, M., Angele, M.K., Huss, R., Günther, C., Nelson, P.J., Bruns, C.J., and Heinemann, V. (2015). Treatment of advanced gastrointestinal tumors with genetically modified autologous mesenchymal stromal cells (TREAT-ME1): study protocol of a phase I/II clinical trial. BMC Cancer *15*, 237. https://doi.org/10.1186/s12885-015-1241-x.

32. Shi, Y., Zhang, J., Li, Y., Feng, C., Shao, C., Shi, Y., and Fang, J. (2025). Engineered mesenchymal stem/stromal cells against cancer. Cell Death Dis. *16*, 113. https://doi.org/10.1038/s41419-025-07443-0.

33. Kitzberger, C., Spellerberg, R., Morath, V., Schwenk, N., Schmohl, K.A., Schug, C., Urnauer, S., Tutter, M., Eiber, M., Schilling, F., et al. (2022). The sodium iodide symporter (NIS) as theranostic gene: its emerging role in new imaging modalities and non-viral gene therapy. EJNMMI Res. *12*, 25. https://doi.org/10.1186/s13550-022-00888-w.

34. Kitzberger, C., Shehzad, K., Morath, V., Spellerberg, R., Ranke, J., Steiger, K., Kälin, R.E., Multhoff, G., Eiber, M., Schilling, F., et al. (2023). Interleukin-6-controlled, mesenchymal stem cell-based sodium/iodide symporter gene therapy improves survival of glioblastoma-bearing mice. Mol. Ther. Oncolytics *30*, 238–253. https://doi.org/10.1016/j.omto.2023.08.004.

35. Kitzberger, C., Spellerberg, R., Han, Y., Schmohl, K.A., Stauss, C., Zach, C., Kälin, R.E., Multhoff, G., Eiber, M., Schilling, F., et al. (2023). Mesenchymal Stem Cell-mediated Image-guided Sodium Iodide Symporter (NIS) Gene Therapy Improves Survival of Glioblastoma-bearing Mice. Clin. Cancer Res. *29*, 930–942. https://doi.org/10.1158/1078-0432.CCR-22-1433.

36. Knoop, K., Schwenk, N., Dolp, P., Willhauck, M.J., Zischek, C., Zach, C., Hacker, M., Göke, B., Wagner, E., Nelson, P.J., and Spitzweg, C. (2013). Stromal targeting of sodium iodide symporter using mesenchymal stem cells allows enhanced imaging and therapy of hepatocellular carcinoma. Hum. Gene Ther. *24*, 306–316. https://doi.org/10.1089/hum.2012.104.

37. Knoop, K., Schwenk, N., Schmohl, K., Müller, A., Zach, C., Cyran, C., Carlsen, J., Böning, G., Bartenstein, P., Göke, B., et al. (2015). Mesenchymal stem cell-mediated, tumor stroma-targeted radioiodine therapy of metastatic colon cancer using the sodium iodide symporter as theranostic gene. J. Nucl. Med. *56*, 600–606. https://doi.org/10.2967/jnumed.114.146662.

38. Muller, A.M., Schmohl, K.A., Knoop, K., Schug, C., Urnauer, S., Hagenhoff, A., Clevert, D.A., Ingrisch, M., Niess, H., Carlsen, J., et al. (2016). Hypoxia-targeted 131I therapy of hepatocellular cancer after systemic mesenchymal stem cell-mediated sodium iodide symporter gene delivery. Oncotarget *7*, 54795–54810. https://doi.org/10.18632/oncotarget.10758.

39. Schug, C., Gupta, A., Urnauer, S., Steiger, K., Cheung, P.F.Y., Neander, C., Savvatakis, K., Schmohl, K.A., Trajkovic-Arsic, M., Schwenk, N., et al. (2019). A Novel Approach for Image-Guided (131)I Therapy of Pancreatic Ductal Adenocarcinoma Using Mesenchymal Stem Cell-Mediated NIS Gene Delivery. Mol. Cancer Res. *17*, 310–320. https://doi.org/10.1158/1541-7786.MCR-18-0185.

40. Schug, C., Kitzberger, C., Sievert, W., Spellerberg, R., Tutter, M., Schmohl, K.A., Eberlein, B., Biedermann, T., Steiger, K., Zach, C., et al. (2019). Radiation-Induced Amplification of TGFB1-Induced Mesenchymal Stem Cell-Mediated Sodium Iodide Symporter (NIS) Gene (131)I Therapy. Clin. Cancer Res. *25*, 5997–6008. https://doi.org/10.1158/1078-0432.CCR-18-4092.

41. Schug, C., Sievert, W., Urnauer, S., Müller, A.M., Schmohl, K.A., Wechselberger, A., Schwenk, N., Lauber, K., Schwaiger, M., Multhoff, G., et al. (2018). External Beam Radiation Therapy Enhances Mesenchymal Stem Cell-Mediated Sodium-Iodide Symporter Gene Delivery. Hum. Gene Ther. *29*, 1287–1300. https://doi.org/10.1089/hum.2018.025.

42. Schug, C., Urnauer, S., Jaeckel, C., Schmohl, K.A., Tutter, M., Steiger, K., Schwenk, N., Schwaiger, M., Wagner, E., Nelson, P.J., and Spitzweg, C. (2019). TGFB1-driven mesenchymal stem cell-mediated NIS gene transfer. Endocr. Relat. Cancer *26*, 89–101. https://doi.org/10.1530/ERC-18-0173.

43. Tutter, M., Schug, C., Schmohl, K.A., Urnauer, S., Kitzberger, C., Schwenk, N., Petrini, M., Zach, C., Ziegler, S., Bartenstein, P., et al. (2021). Regional Hyperthermia Enhances Mesenchymal Stem Cell Recruitment to Tumor Stroma: Implications for Mesenchymal Stem Cell-Based Tumor Therapy. Mol. Ther. *29*, 788–803. https://doi.org/10.1016/j.ymthe.2020.10.009.

44. Tutter, M., Schug, C., Schmohl, K.A., Urnauer, S., Schwenk, N., Petrini, M., Lokerse, W.J.M., Zach, C., Ziegler, S., Bartenstein, P., et al. (2020). Effective control of tumor growth through spatial and temporal control of theranostic sodium iodide symporter (NIS) gene expression using a heat-inducible gene promoter in engineered mesenchymal stem cells. Theranostics *10*, 4490–4506. https://doi.org/10.7150/thno.41489.

45. Spitzweg, C., Nelson, P.J., Wagner, E., Bartenstein, P., Weber, W.A., Schwaiger, M., and Morris, J.C. (2021). The sodium iodide symporter (NIS): novel applications for radionuclide imaging and treatment. Endocr. Relat. Cancer *28*, T193–T213. https://doi.org/10.1530/ERC-21-0177.

46. Von Luttichau, I., Notohamiprodjo, M., Wechselberger, A., Peters, C., Henger, A., Seliger, C., Djafarzadeh, R., Huss, R., and Nelson, P.J. (2005). Human adult CD34- progenitor cells functionally express the chemokine receptors CCR1, CCR4, CCR7, CXCR5, and CCR10 but not CXCR4. Stem Cells Dev *14*, 329–336. https://doi.org/10.1089/scd.2005.14.329.

47. Willhauck, M.J., Sharif Samani, B.R., Gildehaus, F.J., Wolf, I., Senekowitsch-Schmidtke, R., Stark, H.J., Göke, B., Morris, J.C., and Spitzweg, C. (2007). Application of 188rhenium as an alternative radionuclide for treatment of prostate cancer after tumor-specific sodium iodide symporter gene expression. J. Clin. Endocrinol. Metab. *92*, 4451–4458. https://doi.org/10.1210/jc.2007-0402.

48. Willhauck, M.J., Samani, B.R.S., Wolf, I., Senekowitsch-Schmidtke, R., Stark, H.J., Meyer, G.J., Knapp, W.H., Göke, B., Morris, J.C., and Spitzweg, C. (2008). The potential of 211Astatine for NIS-mediated radionuclide therapy in prostate cancer. Eur. J. Nucl. Med. Mol. Imaging *35*, 1272–1281. https://doi.org/10.1007/s00259-008-0775-4.

49. Klutz, K., Willhauck, M.J., Wunderlich, N., Zach, C., Anton, M., Senekowitsch-Schmidtke, R., Göke, B., and Spitzweg, C. (2011). Sodium iodide symporter (NIS)-mediated radionuclide ((131)I, (188)Re) therapy of liver cancer after transcriptionally targeted intratumoral in vivo NIS gene delivery. Hum. Gene Ther. *22*, 1403–1412. https://doi.org/10.1089/hum.2010.158.

50. Landa, I., Pozdeyev, N., Korch, C., Marlow, L.A., Smallridge, R.C., Copland, J.A., Henderson, Y.C., Lai, S.Y., Clayman, G.L., Onoda, N., et al. (2019). Comprehensive Genetic Characterization of Human Thyroid Cancer Cell Lines: A Validated Panel for Preclinical Studies. Clin. Cancer Res. *25*, 3141–3151. https://doi.org/10.1158/1078-0432.CCR-18-2953.

51. Thalmeier, K., and Huss, R. (2001). Highly efficient retroviral gene transfer into immortalized CD34(-) cells and organ distribution after transplantation into NOD/SCID mice. Cytotherapy *3*, 245–251. https://doi.org/10.1080/146532401317070871.

52. Jackel, C., Nogueira, M.S., Ehni, N., Kraus, C., Ranke, J., Dohmann, M., Noessner, E., and Nelson, P.J. (2016). A vector platform for the rapid and efficient engineering of stable complex transgenes. Sci. Rep. *6*, 34365. https://doi.org/10.1038/srep34365.

53. Spitzweg, C., Zhang, S., Bergert, E.R., Castro, M.R., McIver, B., Heufelder, A.E., Tindall, D.J., Young, C.Y., and Morris, J.C. (1999). Prostate-specific antigen (PSA) promoter-driven androgen-inducible expression of sodium iodide symporter in prostate cancer cell lines. Cancer Res. *59*, 2136–2141.

54. Schmohl, K.A., Muller, A.M., Wechselberger, A., Ruhland, S., Salb, N., Schwenk, N., Heuer, H., Carlsen, J., Goke, B., Nelson, P.J., and Spitzweg, C. (2015). Thyroid hormones and tetrac: new regulators of tumour stroma formation via integrin alphavbeta3. Endocr. Relat. Cancer *22*, 941–952. https://doi.org/10.1530/ERC-15-0245.

55. Michl, M., Taverna, F., Kumbrink, J., Schiergens, T.S., Heinemann, V., Engel, J., Kirchner, T., and Neumann, J. (2021). Biomarker alterations associated with distinct patterns of metastatic spread in colorectal cancer. Virchows Arch. *478*, 695–705. https://doi.org/10.1007/s00428-020-02983-6.

56. Spitzweg, C., Baker, C.H., Bergert, E.R., O'Connor, M.K., and Morris, J.C. (2007). Image-guided radioiodide therapy of medullary thyroid cancer after carcinoembryonic antigen promoter-targeted sodium iodide symporter gene expression. Hum. Gene Ther. *18*, 916–924. https://doi.org/10.1089/hum.2007.081.

57. Urnauer, S., Morys, S., Krhac Levacic, A., Müller, A.M., Schug, C., Schmohl, K.A., Schwenk, N., Zach, C., Carlsen, J., Bartenstein, P., et al. (2016). Sequence-defined cMET/HGFR-targeted Polymers as Gene Delivery Vehicles for the Theranostic Sodium Iodide Symporter (NIS) Gene. Mol. Ther. *24*, 1395–1404. https://doi.org/10.1038/mt.2016.95.

YMTHE, Volume *33*

Supplemental Information

TGF-β1-based restoration of sodium iodide symporter expression in radioiodine-refractory differentiated thyroid cancer via engineered MSCs

Yang Han, Viktoria F. Koehler, James Nagarajah, Kathrin A. Schmohl, Christina Stauss, Nathalie Schwenk, Rebekka Spellerberg, Carolin Kitzberger, Joerg Kumbrink, Christian Zach, Katja Steiger, John C. Morris, Wolfgang A. Weber, Peter Bartenstein, Sibylle I. Ziegler, Peter J. Nelson, and Christine Spitzweg

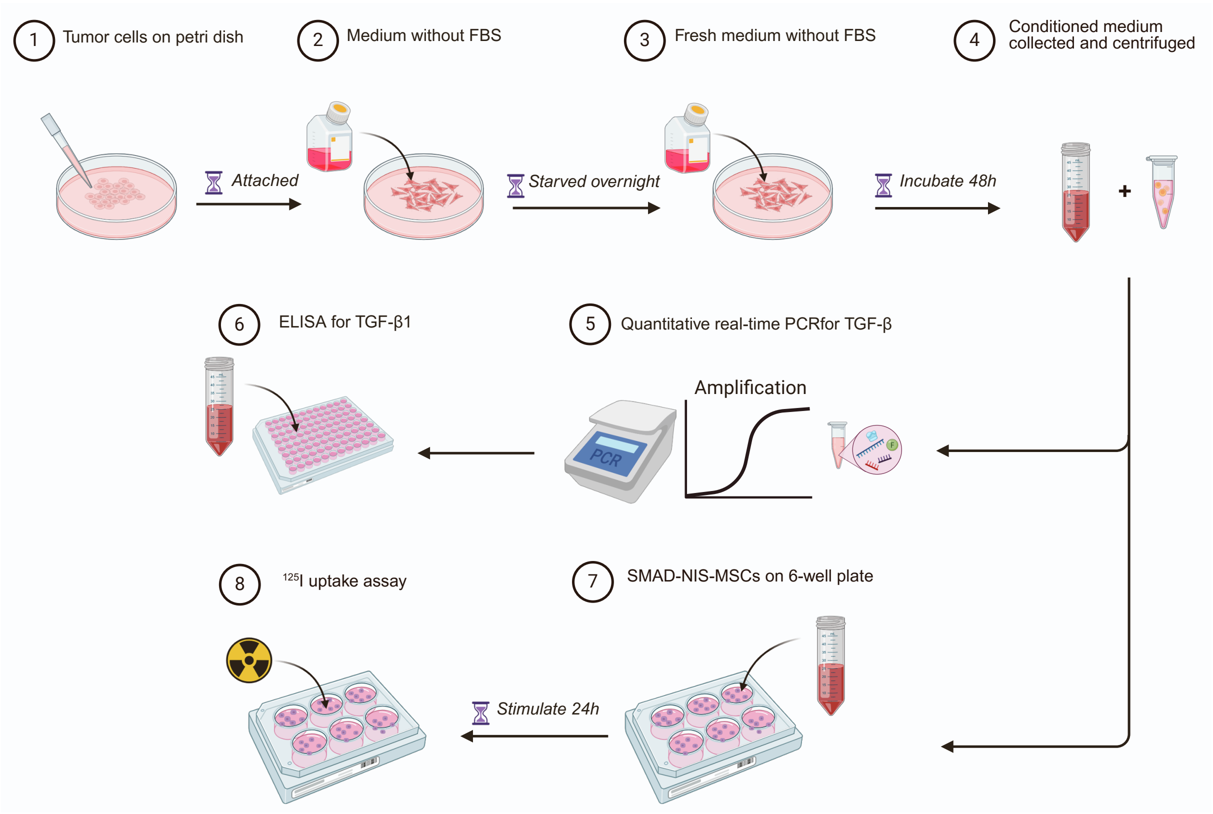

Figure S1. TGF-β expression in thyroid cancer cell lines and stimulation of RAI uptake in SMAD-NIS-MSCs by tumor CM. *Illustration of the experimental design.*

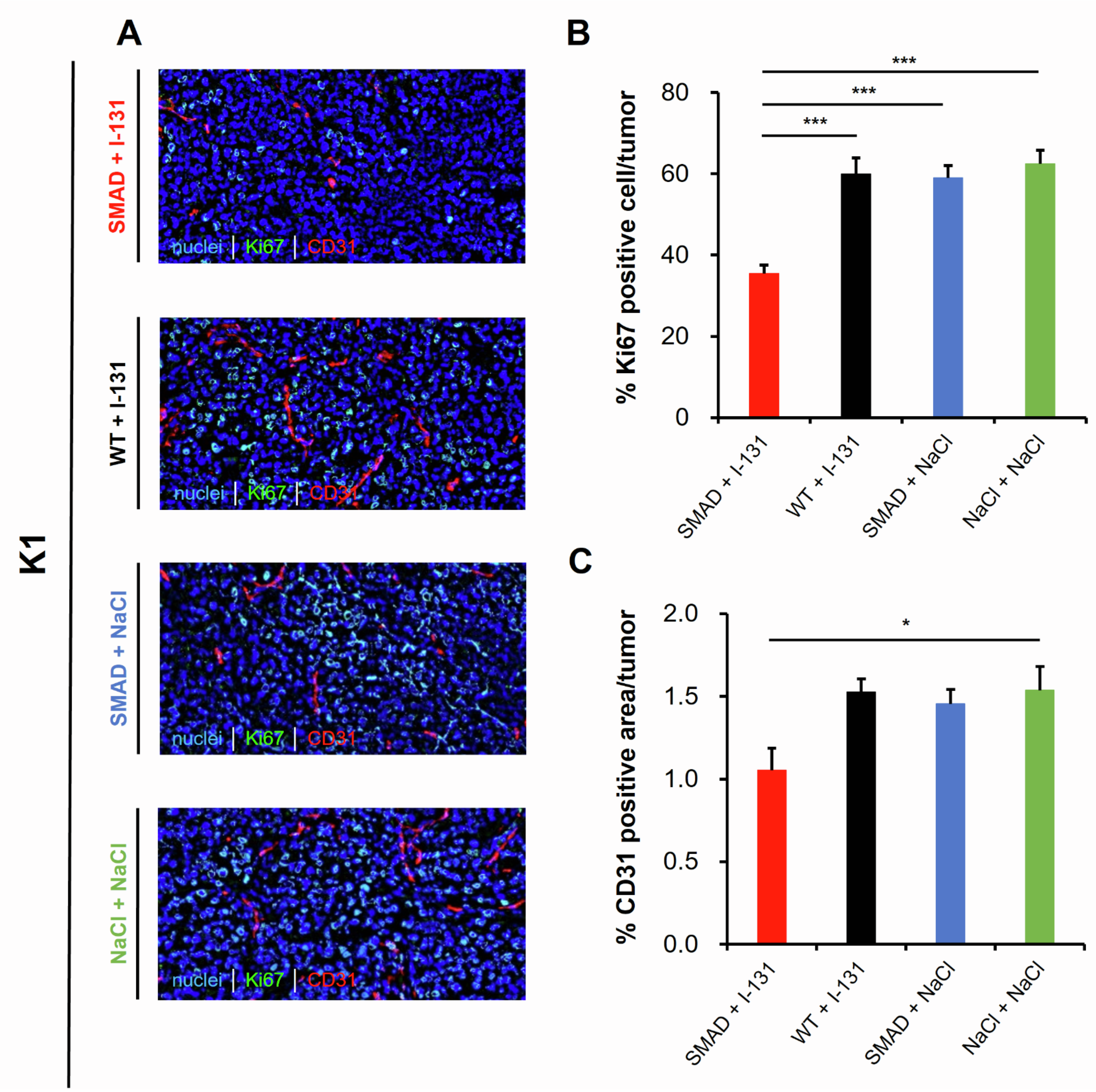
A
B
C
K1
SMAD + I-131
WT + I-131
SMAD + NaCl
NaCl + NaCl
nuclei | Ki67 | CD31
% Ki67 positive cell/tumor
% CD31 positive area/tumor
***
*
0
20
40
60
80
0.0
0.5
1.0
1.5
2.0

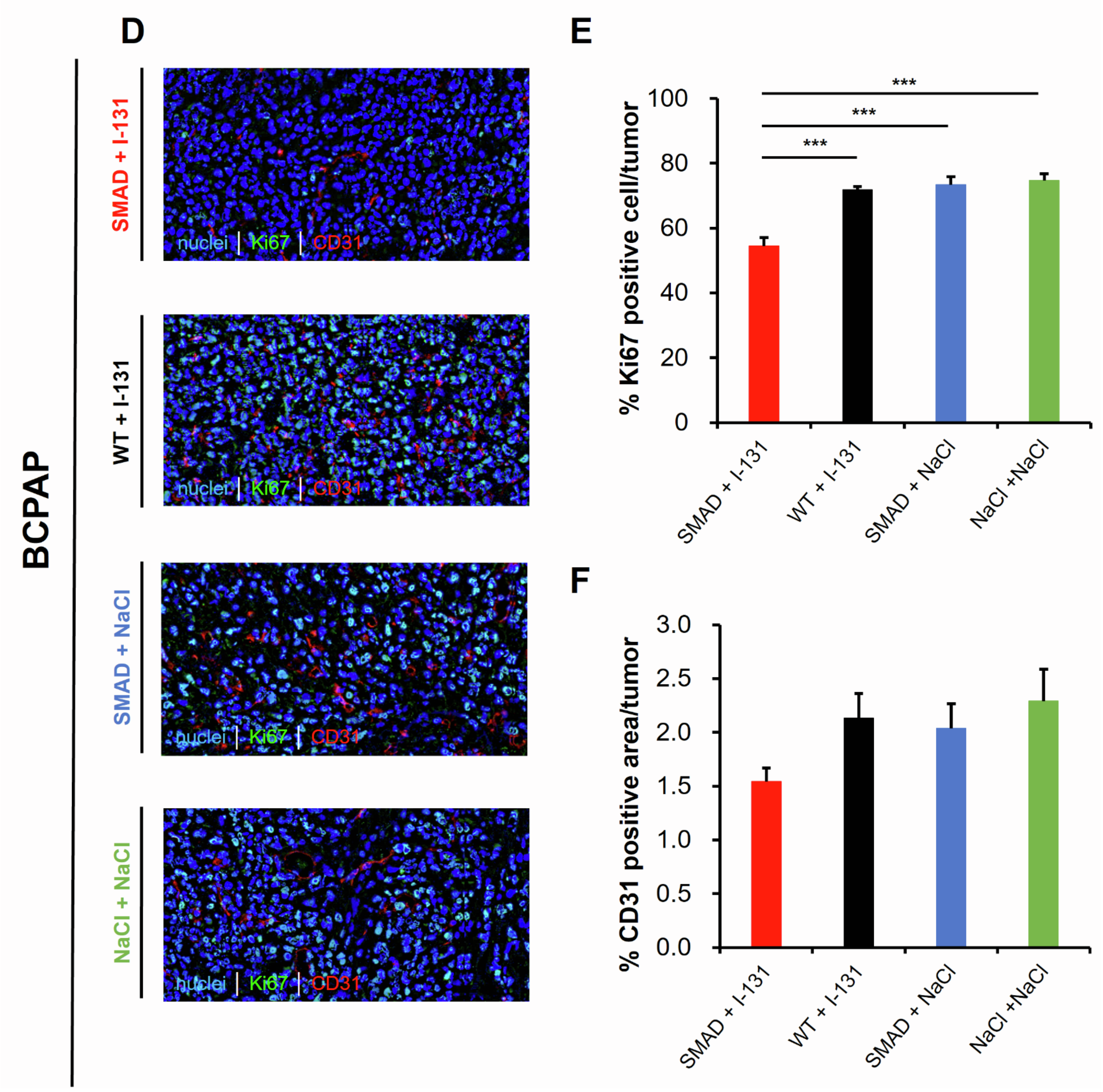

Figure S2. Ex vivo analysis of tumor cell proliferation and vascularization. *Tumor cell proliferation was assessed by Ki67 immunofluorescence staining (green) and blood vessel density by CD31 staining (red) on frozen tumor sections* ***(A, D)****. The staining revealed significantly decreased proliferation as well as reduced blood vessel density in K1 tumors* ***(B, C)*** *and BCPAP tumors* ***(E, F)*** *treated with SMAD-NIS-MSCs*

*followed by ^{131}I treatment as compared to the control tumors (mean ± SEM; *p<0.05, ***p<0.001). One representative image is shown each (20× magnification).*
